# Lipid binding by the N-terminal motif mediates plasma membrane localization of *Bordetella* effector protein BteA

**DOI:** 10.1016/j.jbc.2021.100607

**Published:** 2021-03-28

**Authors:** Ivana Malcova, Ladislav Bumba, Filip Uljanic, Darya Kuzmenko, Jana Nedomova, Jana Kamanova

**Affiliations:** 1 Laboratory of Infection Biology, Institute of Microbiology of the Czech Academy of Sciences, Prague, Czech Republic; 2 Laboratory of Molecular Biology of Bacterial Pathogens, Institute of Microbiology of the Czech Academy of Sciences, Prague, Czech Republic

**Keywords:** lipid–protein interaction, membrane localization domain, plasma membrane, protein motif, surface plasmon resonance, *Saccharomyces cerevisiae*, virulence factor, type III secretion system, BteA effector protein, *Bordetella pertussis*, 4HBM, four-helix bundle membrane localization domain, GST, glutathione-*S*-transferase, LDH, lactate dehydrogenase, LRT domain, lipid raft targeting domain, MLD, membrane localization domain, MOI, multiplicity of infection, PA, phosphatidic acid, PC, phosphatidylcholine, PIP2, phosphatidylinositol 4,5-bisphosphate, PS, phosphatidylserine, SPR, surface plasmon resonance, T3SS, type III secretion system

## Abstract

The respiratory pathogens *Bordetella pertussis* and *Bordetella bronchiseptica* employ a type III secretion system (T3SS) to inject a 69-kDa BteA effector protein into host cells. This effector is known to contain two functional domains, including an N-terminal lipid raft targeting (LRT) domain and a cytotoxic C-terminal domain that induces nonapoptotic and caspase-1–independent host cell death. However, the exact molecular mechanisms underlying the interaction of BteA with plasma membrane (PM) as well as its cytotoxic activity in the course of *Bordetella* infections remain poorly understood. Using a protein–lipid overlay assay and surface plasmon resonance, we show here that the recombinant LRT domain binds negatively charged membrane phospholipids. Specifically, we determined that the dissociation constants of the LRT domain–binding liposomes containing phosphatidylinositol 4,5-bisphosphate, phosphatidic acid, and phosphatidylserine were ∼450 nM, ∼490 nM, and ∼1.2 μM, respectively. Both phosphatidylserine and phosphatidylinositol 4,5-bisphosphate were required to target the LRT domain and/or full-length BteA to the PM of yeast cells. The membrane association further involved electrostatic and hydrophobic interactions of LRT and depended on a leucine residue in the L1 loop between the first two helices of the four-helix bundle. Importantly, charge-reversal substitutions within the L1 region disrupted PM localization of the BteA effector without hampering its cytotoxic activity during *B. bronchiseptica* infection of HeLa cells. The LRT-mediated targeting of BteA to the cytosolic leaflet of the PM of host cells is, therefore, dispensable for effector cytotoxicity.

Bacterial toxins and effectors localize to specific compartments within the host cell environment to access their intracellular targets and enhance the signaling specificity and efficacy. Various membrane-targeting strategies have evolved to trap the catalytic domain or adaptor domain at the proximity of the membrane-anchored substrates (1). Some of the proteins insert directly into the membrane as integral membrane proteins through transmembrane domains, such as the *Salmonella* type III secretion system (T3SS) effector SteD (2). Others undergo covalent lipid modification, such as *Salmonella* T3SS effector proteins SspH2 and SseI that are S-palmitoylated on a conserved cysteine residue within their N-terminal domains by a specific subset of host–cell palmitoyltransferases (3). Nevertheless, a significant part of bacterial toxins and effectors possess a dedicated membrane localization domain (MLD) that binds phospholipids. For example, recruitment of the *Legionella pneumophila* effector DrrA to the *Legionella*-containing vacuole, where it AMPylates Rab1, is mediated by a phosphatidylinositol 4-phosphate (PI(4)P)-binding domain. This domain is characterized by a deep electropositive binding pocket and surrounding membrane-penetrating leucine residues (4, 5). Another domain termed 4HBM for four-helix bundle MLD is shared by multiple bacterial toxins, including *Pasteurella multocida* mitogenic toxin, multifunctional-autoprocessing RTX toxins, and large clostridial glucosyltransferase toxins exemplified by *Clostridium difficile* toxin A and toxin B and *Clostridium sordellii* lethal toxin (TcsL) (1, 6, 7, 8). The phospholipid-binding site of 4HBM is located at the apex of the structure, the so-called bundle tip, which is formed by two protruding loops, loop 1 (L1) connecting helices 1 and 2, and loop 3 (L3) in between helices 3 and 4. Indeed, the positively charged and hydrophobic residues within L1 and L3 are key for lipid-binding and membrane localization of 4HBM as revealed by their mutagenesis (8, 9). Furthermore, the proper localization of *Pasteurella multocida* mitogenic toxin and TcsL toxins also proved to be critical for both toxin activities and TcsL cytotoxicity (6, 10).

The cytotoxic effector BteA is injected into the host cells by a T3SS of classical *Bordetella* species, *Bordetella pertussis*, and *Bordetella bronchiseptica* (11). These bacteria colonize the ciliated epithelia of the respiratory tract of diverse mammals and cause respiratory illness with differing symptoms, duration, and severity. The strictly human-adapted *B. pertussis* is the primary causative agent of pertussis or whooping cough, a contagious respiratory illness of humans that remains one of the least controlled vaccine-preventable infectious diseases (12). The *B. bronchiseptica* species, on the other hand, infects a broad range of mammals and causes infections ranging from lethal pneumonia to asymptomatic and chronic respiratory carriage (12, 13). The activity of T3SS of *B. bronchiseptica* is required for persistent colonization of the lower respiratory tract of rats, mice, and pigs, presumably because of the actions of BteA effector (14, 15, 16). In tissue culture, however, BteA actions account for rapid cell death that is nonapoptotic and caspase-1 independent (17). Remarkably, compared with the high BteA–mediated cytotoxicity of *B. bronchiseptica*, the cytotoxicity of BteA of *B. pertussis* is strongly attenuated because of the insertion of an extra alanine at position 503, which may represent an evolutionary adaptation of *B. pertussis* (18). Nevertheless, the role of BteA and T3SS activity in the pathophysiology of human pertussis remains to be established.

The 69-kDa effector protein BteA exhibits modular architecture, consisting of two functional domains, an N-terminal localization/lipid raft targeting domain of ∼130 amino acid residues and a cytotoxic C-terminal domain of ∼528 amino acid residues without any known structural homologs (19). The N-terminal domain of BteA is sufficient to target GFP to lipid rafts of HeLa cell plasma membrane (PM) and was therefore termed lipid raft targeting (LRT) domain (19). Homologous domains were identified by sequence similarity searches in a number of known and putative virulence factors of other bacteria, including a T3SS effector Plu4750 and a multifunctional-autoprocessing RTX toxin Plu3217 of *Photorhabdus luminescens* which as LRT targeted GFP to lipid rafts (19). The structure of LRT domain, described by LRT helices (A-A’)-B-C-E, displays an overall tertiary fold similar to 4HBM (20, 21). Interestingly, the topology of the LRT domain tip is different from that of 4HBM, being formed by the loop region L1, connecting helices A and B, and the capping perpendicular helix D, connecting helices C and E (21). The *in vivo* membrane-targeting mechanism of LRT domain and its contribution to the cytotoxicity of BteA effector remains poorly characterized. The recombinant LRT domain binds phosphatidylinositol 4,5-bisphosphate (PIP2)-containing nanodisks suggesting that BteA may associate with the host PM and lipid rafts through phospholipid interaction (21). Interestingly, ectopically expressed BteA of *B. bronchiseptica* remains cytotoxic even upon deletion of the LRT domain or the removal of the first 200 N-terminal amino acids, while its cytotoxicity is diminished upon deletion of the last 14 C-terminal amino acid residues (19, 22).

In this work, we provide a mechanical insight into *in vivo* membrane targeting of the LRT domain and its contribution to BteA cytotoxicity during *B. bronchiseptica* infection.

## Results

### The specificity of the phospholipid binding by LRT motif of BteA effector

**Figure 1:**
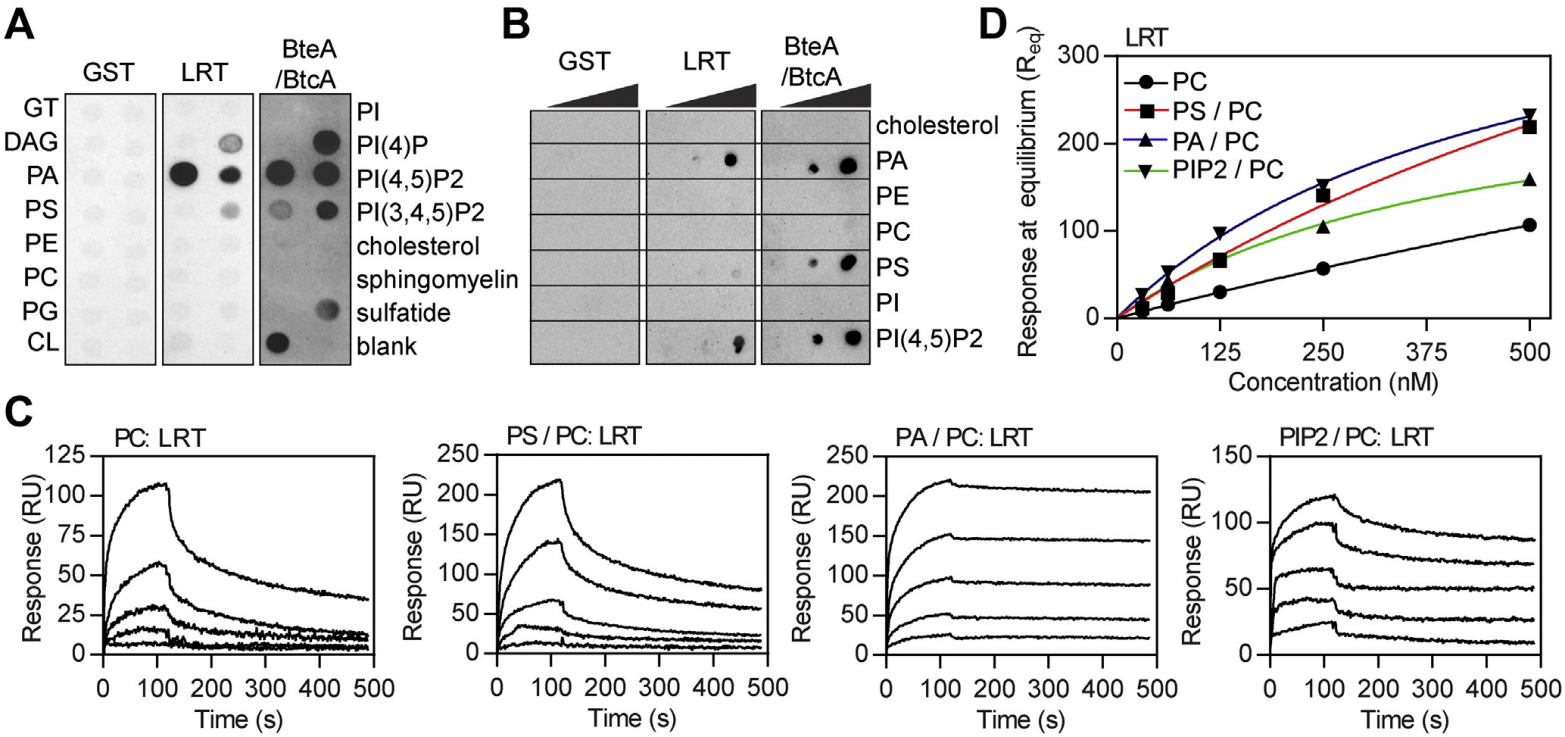
**Phospholipid binding by the N-terminal motif of *Bordetella* effector BteA.***A* and *B*, protein–lipid overlay assay. The recombinant GST-tagged N-terminal LRT domain (LRT) and full-length BteA (BteA/BtcA) protein of *B. pertussis* were incubated at 5 μg/ml with commercial (*A*) or home-made (*B*) lipid arrays. The binding was detected using an anti-GST antibody followed by chemiluminescence detection. Recombinant GST was used as a control. *C*, SPR real-time kinetics of LRT binding to lipid membranes. Serially diluted LRT protein (at 500, 250, 125, 62.5, and 31.25 nM concentrations) was injected in parallel over the neutravidin sensor chip coated with the immobilized liposomes (100 nm in diameter) containing PC, PS/PC (20:80), PA/PC (5:95), or PC/PIP2 (5:95) and left to associate (120 s) and dissociate (380 s) at constant flow rate of 30 μl/min. The sensograms show the representative binding curves from five independent “one-shot kinetic” experiments. *D*, SPR steady-state analysis of LRT binding to lipid vesicles. Serially diluted LRT protein was injected over the immobilized lipid vesicles to reach the SPR binding equilibrium, and the near-equilibrium values (R_eq_) were plotted against LRT concentrations (P_0_). Solid lines represent binding isotherms determined by a nonlinear fitting of the data using the equation R_eq_ = R_max_/(1 + K_D_/P_0_). CL, cardiolipin; DAG, diacylglycerol; GT, triglyceride; GST, glutathione-S-transferase; LRT, lipid raft targeting; PA, phosphatidic acid; PC, phosphatidylcholine; PG, phosphatidylglycerol; PE, phosphatidylethanolamine; PI, phosphatidylinositol; PIP, PIP2, PIP3, phosphatidylinositol phosphates; PS, phosphatidylserine; SPR, surface plasmon resonance.

To understand the membrane-targeting mechanism of BteA effector, the lipid-binding properties of its N-terminal LRT domain comprising 130 amino acid residues were tested in a protein–lipid overlay assay. The purified recombinant glutathione-*S*-transferase (GST)-tagged LRT domain of *B. pertussis* BteA (LRT) was used to probe commercial lipid strips. These have been spotted with 100 pmol of different lipids, and bound LRT was detected using an anti-GST antibody. In contrast to GST alone, which displayed no binding affinity for lipids, LRT protein preferentially interacted with negatively charged lipids, with a preference for PIP2 and phosphatidic acid (PA), as shown in Figure 1*A* and Fig. S1*A*. The LRT binding depended on the spotted lipid concentration as further corroborated using home-made lipid arrays spotted with a concentration gradient of 10, 100, and 1000 pmol of various lipids or cholesterol per spot (Fig. 1*B*). Interestingly, as also shown in Figure 1, *A* and *B* and Fig. S1*A*, the GST-tagged full-length BteA protein of *B. pertussis* in a complex with its cognate chaperone BtcA (BteA/BtcA) exhibited similar, although, somehow stronger binding of negatively charged lipids than LRT alone. Nevertheless, the lipid-binding abilities of the full-length BteA protein might have been affected by the presence of BtcA, which co-purified with the BteA. In contrast to LRT protein, we were unable to produce soluble BteA without its cognate chaperone BtcA in *Escherichia coli*.

To avoid the limitations of the protein–lipid overlay assay and experiment-to-experiment variability, we next analyzed LRT interaction with a native phospholipid bilayer by surface plasmon resonance (SPR). Four types of large unilamellar vesicles consisting of (i) phosphatidylcholine (PC) only, or the mixture of (ii) phosphatidylserine with PC (PS/PC, molar ratio 20:80), (iii) PA with PC (PA/PC, molar ratio 5:95), and (iv) PIP2 with PC (PIP2/PC, molar ratio 5:95) were prepared and captured on a neutravidin-coated sensor chip. The recombinant proteins were then serially diluted and injected over the immobilized liposome surface to monitor their binding. While GST alone did not interact with any of the lipid vesicles (Fig. S1*B*), the concentration-dependent binding curves of LRT protein revealed its interaction with all four membrane surfaces with the typical association and dissociation phases of the sensograms (Fig. 1*C*). The binding affinities of LRT to lipid vesicles were next calculated from steady-state binding data as the global fitting of the binding curves to several kinetic models did not provide satisfactory results in terms of χ^2^ and residual statistics. The near-equilibrium values (R_eq_), which were taken from the end of the association phase of the individual binding curves, were plotted as a function of the LRT concentration (Fig. 1*D*), and the equilibrium dissociation constant (K_D_) was determined by nonlinear least-squares analysis of the binding isotherm. As shown in Figure 1*D*, the binding of LRT to PC vesicles was not saturable (up to 500 nM), indicating a nonspecific interaction of the LRT with the charge-neutral phospholipid bilayer. In contrast, the negatively charged lipid surfaces conferred the binding of LRT in a saturable manner, with apparent K_D_ values of 1225 ± 245 for PS-enriched vesicles and 492 ± 86 nM and 446 ± 65 nM for PA- and PIP2-containing vesicles, respectively. These results, thus, demonstrate that the LRT motif has a direct affinity for negatively charged lipid surfaces *in vitro*.

### Phospholipids PS and PIP2 mediate plasma membrane localization of BteA effector

It was next important to test for the role of negatively charged lipids in guiding the localization of LRT and full-length BteA effector *in vivo*. To this end, the cellular distribution of ectopically expressed GFP-tagged LRT and BteA proteins of *B. pertussis* was analyzed by live-cell fluorescence microscopy in *Saccharomyces cerevisiae* WT and mutant strains that harbored specific defects in biosynthesis pathways for PS, PA, and PIP2.

**Figure 2:**
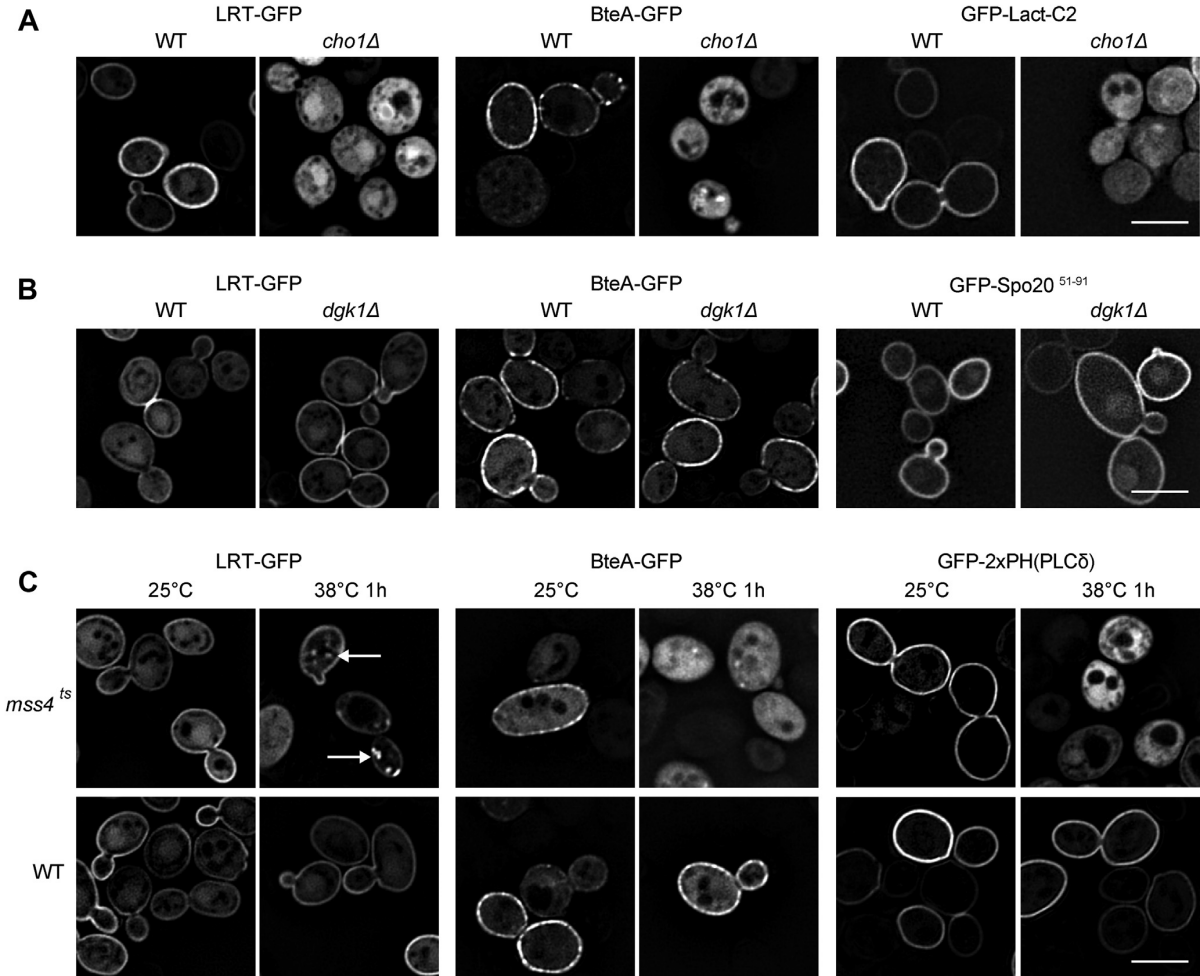
**Phospholipids PS and PIP2 guide plasma membrane association of *Bordetella* effector BteA and its LRT motif *in vivo*.** Localization of GFP-tagged LRT domain (LRT–GFP) and full-length BteA (BteA–GFP) of *B. pertussis* was analyzed after galactose induction in the following *S. cerevisiae* strains. *A*, wildtype *S. cerevisiae* BY4742 (WT) and its *cho1*Δ derivative deficient in PS synthesis. *B*, wildtype *S. cerevisiae* BY4741 (WT) and its *dgk1*Δ derivative deficient in PA synthesis. *C*, wildtype *S. cerevisiae* SEY6210 (WT) and its temperature-sensitive *mss4*^*ts*^ derivative exhibiting depleted levels of PIP2 after the shift from permissive (25 °C) to restrictive temperature (38 °C). *White arrows* indicate the relocalization of the LRT–GFP protein from the plasma membrane to internal puncta in the *mss4*^*ts*^ mutant. The expression of the GFP-tagged phospholipid probes GFP-Lact-C2 (PS-specific probe), GFP-Spo20^51–91^ (PA-specific probe), and GFP-2xPH(PLCδ) (PIP2-specific probe) was used to visualize cellular PS, PA, and PIP2 levels, respectively. No difference in the localization of PA probe was observed in the *dgk1*Δ mutant (see text for further details). Representative images from two independent experiments with the same outcome are presented. Scale bar, 5 μm. LRT, lipid raft targeting; PA, phosphatidic acid; PIP2, phosphatidylinositol 4,5-bisphosphate; PS, phosphatidylserine.

As illustrated in Figure 2*A*, when LRT–GFP was expressed in WT strain of *S. cerevisiae* BY4742, it readily associated with the PM, similarly to its previously reported localization in HeLa cells (19). The full-length BteA–GFP protein also exhibited peripheral localization in WT strain, although its distribution was patchier (Fig. 2*A*). Upon expression in the *cho1*Δ mutant harboring a deletion of PS synthase Cho1, however, both proteins were distributed throughout the cytoplasm without any PM localization (Fig. 2*A*). In the same manner, GFP-tagged PS-specific binding protein, GFP-Lact-C2, localized to the PM of WT strain and dispersed throughout the cytoplasm in the *cho1*Δ mutant (Fig. 2*A*). In contrast, no difference in the localization of PIP2-specific binding protein tagged with GFP, GFP-2xPH(PLCδ), was observed between both strains (Fig. S2*A*). These results demonstrate that both LRT and BteA proteins respond specifically to a decreased level of PS in the *cho1*Δ mutant and point to the importance of PS in their localization.

To address the role of PA, LRT–GFP and full-length BteA–GFP proteins were expressed in the *dgk1*Δ mutant of *S. cerevisiae*, which harbors a deletion of diacylglycerol kinase responsible for PA synthesis from diacylglycerol (23). As shown in Figure 2*B*, however, no change in the localization of analyzed proteins or commonly used GFP-tagged PA sensor, GFP-Spo20^51–91^ (24), was observed between the *dgk1*Δ mutant and parental WT strain BY4741 of *S. cerevisiae*. These data suggest no depletion of PA levels in the *dgk1*Δ mutant in the used conditions and/or reduced specificity of the commonly used PA sensor.

To test for the role of PIP2, a thermosensitive mutant of the phosphatidylinositol 4-phosphate 5-kinase Mss4 (*mss4*^*ts*^), which generates PIP2 from PI(4)P was used. To confirm the decreased PM levels of PIP2 in the *mss4*^*ts*^ mutant at the restrictive temperature, PIP2 levels were visualized by GFP-2xPH(PLCδ). As shown in Figure 2*C*, GFP-2xPH(PLCδ), indeed, relocated from the PM to cell cytosol after the shift to the restrictive temperature (38 °C, 1 hod). In contrast, the localization of PS-specific probe, GFP-Lact-C2, was unaltered, showing that depletion of PIP2 did not merely disrupt the PM integrity (Fig. S2*B*). As further shown in Figure 2*C*, LRT-GFP and BteA-GFP proteins localized to the PM of the *mss4*^*ts*^ strain at the permissive temperature (25 °C), although the association of BteA–GFP was poor when compared with the parental WT strain SEY6210 (see Fig. S2*C* for comparison at lower magnification). Importantly, at the restrictive temperature (38 °C), BteA-GFP was entirely cytoplasmic localized (Fig. 2*C* and Fig. S2*C*), whereas LRT–GFP partially relocated from the PM to the internal puncta, as highlighted by the white arrows in Figure 2*C*. Collectively, although the contribution of PA to localization of BteA effector *in vivo* is unclear, our data demonstrate that PS and PIP2 levels influence the proper localization of LRT and BteA proteins.

### Definition of structural determinants of the LRT motif in membrane interaction

**Figure 3:**
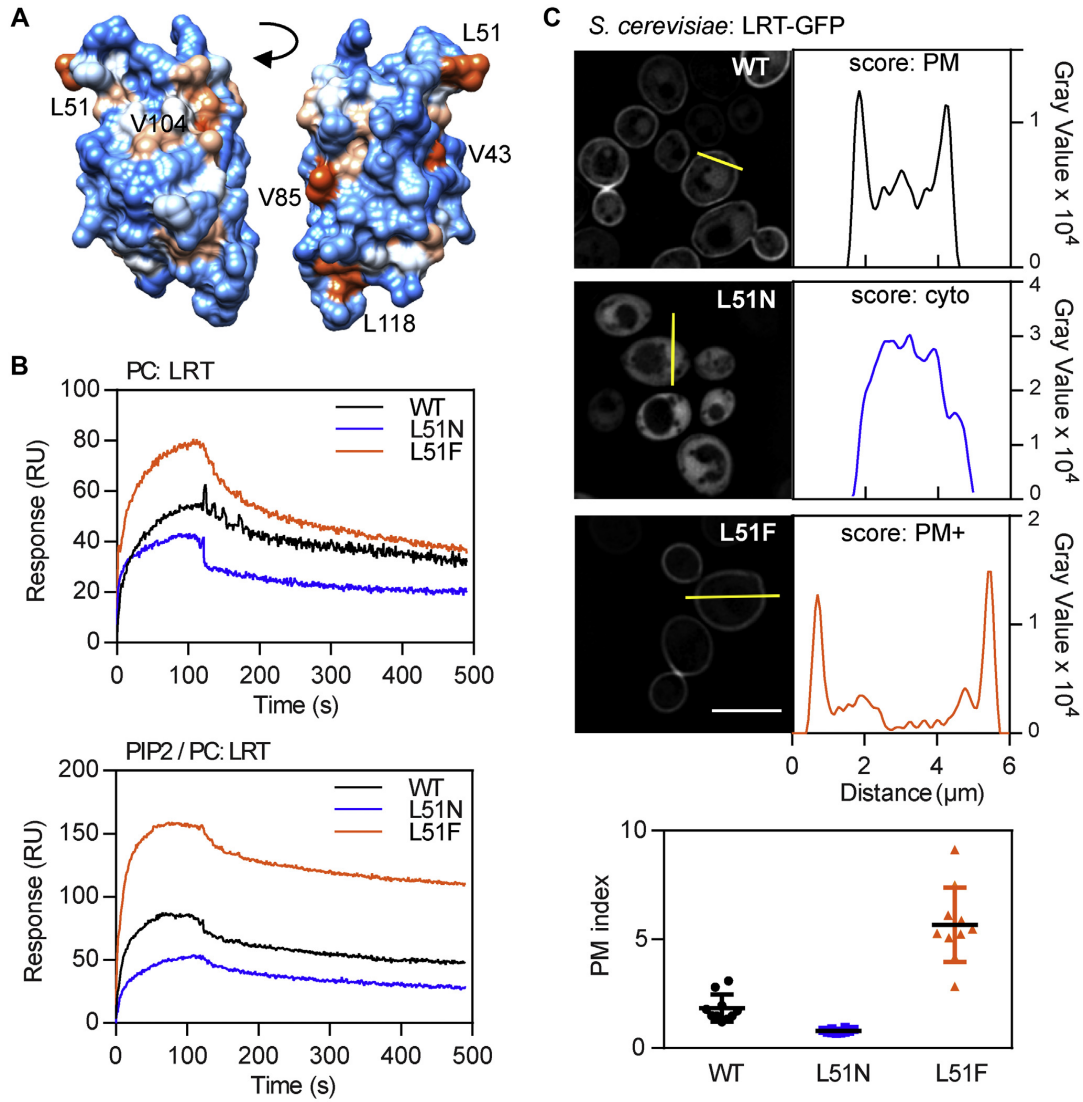
**Leu51 residue is involved in hydrophobic interactions of the LRT motif with a phospholipid membrane.***A*, surface representation of the LRT structure (aa 29–121, PDB code: 6RGN) colored according to the hydrophobicity and visualized by Chimera 1.14rc. The color gradient ranges from *red* for the most hydrophobic to *white* at 0.0 and *blue* for the most hydrophilic. The exposed hydrophobic residues are indicated. *B*, overlay plot of SPR sensograms of the interaction between LRT variants and lipid membranes. Wildtype LRT (WT), LRT-L51N, and LRT-L51F proteins at 250 nM concentration were injected over the neutravidin sensor chip coated with the immobilized lipid vesicles containing PC or PIP2/PC (5:95). The binding curves are representative of five independent “one-shot kinetic” experiments. *C*, localization of GFP-fused LRT variants in *S. cerevisiae* BY4741. Yeast cells with plasmids encoding WT or mutant L51N and L51F LRT–GFP fusion proteins were grown for 20 h in galactose-containing media to induce protein expression. Representative images from two independent experiments with the same outcome are presented. Graphs next to the fluorescence micrographs represent a fluorescence intensity profile along the yellow bar that was used for calculation of a protein plasma membrane (PM) index. See Experimental procedures for details. Values of PM indexes from ten different cells with their mean ± SD are plotted at the bottom of the image for each construct. Scale bar, 5 μm. LRT, lipid raft targeting; PIP2, phosphatidylinositol 4,5-bisphosphate; SPR, surface plasmon resonance.

Having shown that negatively charged phospholipids PS and PIP2 guide the localization of BteA effector *in vivo*, we further analyzed the structural determinants of LRT that confer its membrane interaction. The LRT protein bound to lipid vesicles composed of PC only (Fig. 1*C*), suggesting that it associates with lipid membranes at least partly *via* hydrophobic interactions. Indeed, as depicted in Figure 3*A*, several hydrophobicity patches, including a protruding hydrophobic leucine 51 (L51) residue, can be visualized on the surface of the LRT structure (aa 29–121, PDB code: 6RGN, (21)). To test for the role of the L51 residue in the binding of LRT to lipid membranes, the residue was replaced by asparagine (L51N) or phenylalanine (L51F), and the capacity of these mutants to interact with lipidic surfaces was evaluated *in vitro* by SPR and *in vivo* by fluorescence microscopy. As shown in Figure 3*B* and Fig. S3*A*, the substitution of hydrophobic L51 by hydrophilic asparagine decreased the binding of LRT–L51N protein to phospholipid bilayers, regardless of the charge and composition of the immobilized lipid vesicles. In contrast, the replacement of the hydrophobic side chain of L51 by a more hydrophobic aromatic ring of phenylalanine augmented the lipid-binding of LRT–L51F mutant protein. The importance of the L51 hydrophobic side chain in membrane targeting was further corroborated *in vivo* by fluorescence microscopy. As shown in Figure 3*C*, the L51N variant of LRT–GFP fusion protein was distributed throughout the cytoplasm upon ectopic expression in *S. cerevisiae* cells. In contrast, the WT protein preferentially localized to the cell periphery. Besides, the introduction of L51F mutation into LRT–GFP fusion protein enhanced its peripheral localization (Fig. 3*C*). The change of localization for both mutant proteins proved to be significant (*p* < 0.0001, unpaired two-tailed *t* test, n = 10, compared with LRT-WT) as determined by PM indexes of the proteins. These were calculated as the ratios of the highest intensity value measured at the cell periphery and cell interior, which yielded 1.8 ± 0.6 for LRT-WT, and 0.8 ± 0.1 and 5.7 ± 1.7 for LRT–L51N and LRT–51F mutant proteins, respectively (see Experimental procedures for more details) (Fig. 3*C*). Importantly, as shown in Fig. S3*B* by immunoblot analysis, the L51 substitutions within the LRT segment did not affect the GFP-fusion protein stability in yeast, and thus, the change in LRT localization was not related to protein degradation. These data, therefore, demonstrate that the hydrophobic moiety of the L51 residue is required for efficient interaction of LRT with the phospholipid bilayer.

**Figure 4:**
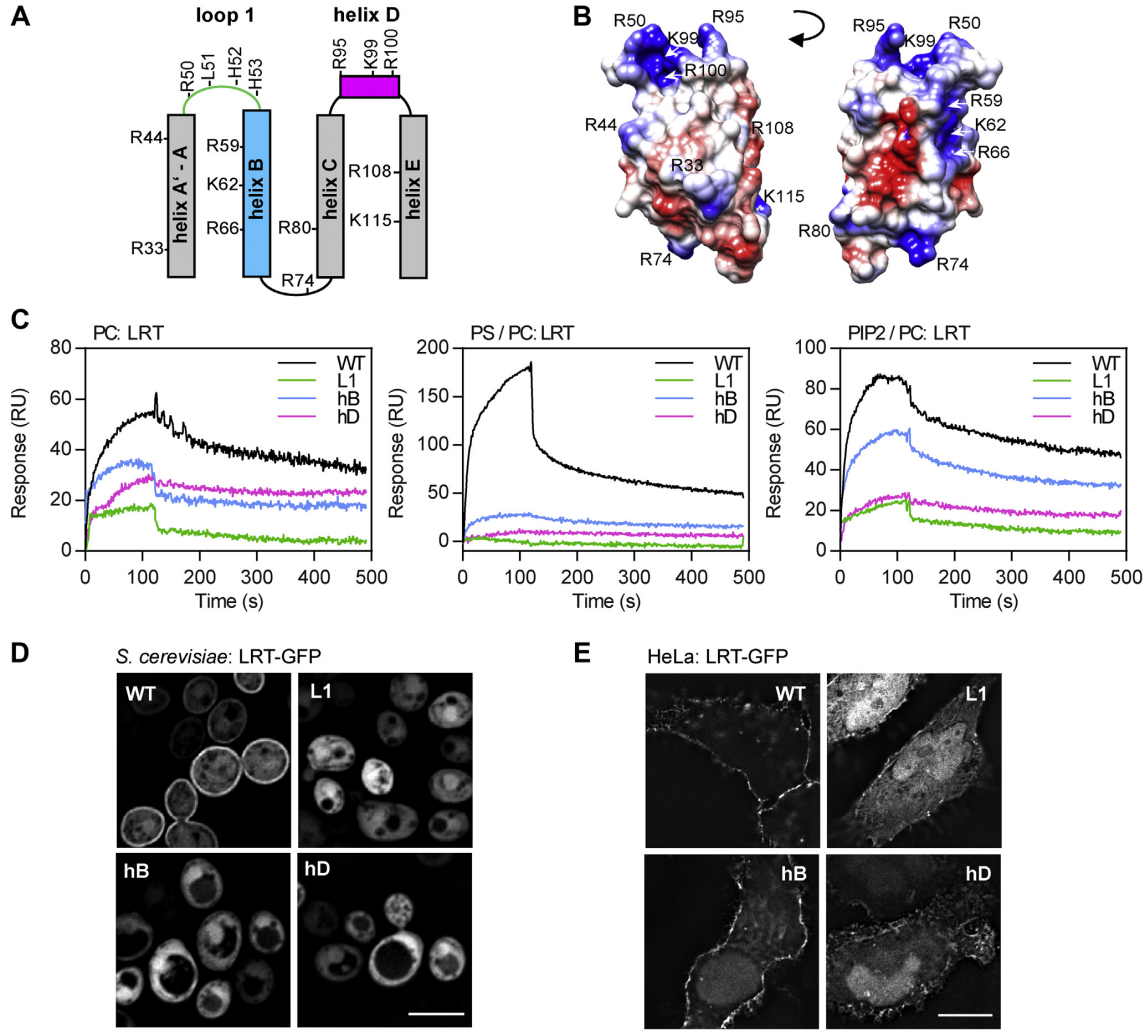
**Electrostatic interactions determine the membrane interaction of the LRT domain.***A* and *B*, topology diagram (*A*) and coulombic surface coloring (*B*) of the LRT helix bundle fold (aa 29–121, PDB code: 6RGN) with the indication of positively charged arginine and lysine residues. The color gradient of coulombic surface coloring, as calculated by Chimera 1.14rc, ranges from the positive charge in *blue* to the negative charge in *red*. *C*, overlay plot of SPR sensograms of the interaction between LRT protein variants and lipid membranes. Wildtype LRT (WT) and its charge-reversal substitution L1 (R50E + H52E + H53E), hB (R59E + K62E + R66E), and hD (K99E + R100E) variants were injected at 250 nM concentration over the neutravidin sensor chip coated with the immobilized lipid vesicles containing PC, PS/PC (20:80), or PIP2/PC (5:95). The sensograms show the representative binding curves obtained from six independent “one-shot kinetic” experiments. *D* and *E*, localization of the GFP-tagged LRT protein variants. *D*, *S. cerevisiae* BY4741 cells harboring plasmids encoding the indicated LRT–GFP protein variants were induced for 20 h for protein expression and examined by live-cell imaging. Scale bar, 5 μm. *E*, HeLa cells were transiently transfected with plasmids expressing the indicated GFP-tagged LRT proteins, fixed after 18 h, and examined by fluorescence microscopy. Scale bar, 20 μm. Representative images from two independent experiments with the same outcome are presented. hB, helix B; hD, helix D; L1, loop1; LRT, lipid raft targeting; PC, phosphatidylcholine; PIP2, phosphatidylinositol 4,5-bisphosphate; PS, phosphatidylserine; SPR, surface plasmon resonance.

We reasoned that besides LRT–L51 hydrophobic interaction with lipid membrane, also electrostatic interactions with negatively charged phospholipid headgroups contribute to membrane binding. Indeed, the structure of LRT revealed a four-helix bundle protein that consists of a large number of positively charged arginine and lysine amino acid residues (Fig. 4, *A* and *B*). To determine critical amino acid residues, a set of LRT mutant proteins harboring charge-reversal substitutions within loop L1 (R50E + H52E + H53E, LRT-L1), helix B (R59E + K62E + R66E, LRT-hB), and helix D (K99E + R100E, LRT-hD) was first constructed, and the capacity of the mutant proteins to interact with the immobilized lipid vesicles was evaluated by SPR. As shown in Figure 4*C*, the LRT–L1 and LRT–hD mutant proteins exhibited almost complete loss of binding to lipid membranes, regardless of their composition. In contrast, the interaction of LRT–hB with the immobilized PIP2-enriched vesicles was only slightly reduced while completely abolished for the PS-containing liposomes. Hence, the positively charged residues within the loop L1 and helices B and D are required for the interaction with negatively charged membranes, whereas the positively charged side chains at the tip of the LRT structure seem to confer the specificity for PIP2.

To corroborate these data *in vivo*, the localization of mutant GFP-tagged LRT proteins was analyzed by fluorescence microscopy in *S. cerevisiae* and HeLa cells upon LRT ectopic expression. As shown in Figure 4*D*, charge reversal substitutions within loop L1 (R50E + H52E + H53E, LRT-GFP-L1), helix B (R59E + K62E + R66E, LRT-GFP-hB), and helix D (K99E + R100E, LRT-GFP-hD) resulted in cytoplasmic localization of the mutant proteins in *S. cerevisiae* cells. In transiently transfected HeLa cells, as compared with LRT–GFP, the mutant proteins also predominantly localized to cell cytosol and accumulated in cell nuclei by passive diffusion through nuclear pore complexes (Fig. 4*E*), although slight PM localization of LRT–GFP–hB was still noticeable. Again, no change in GFP-fusion protein stability was observed in yeast and HeLa cells, as revealed by immunoblot analysis and shown in Fig. S4, *A* and *B*.

**Figure 5:**
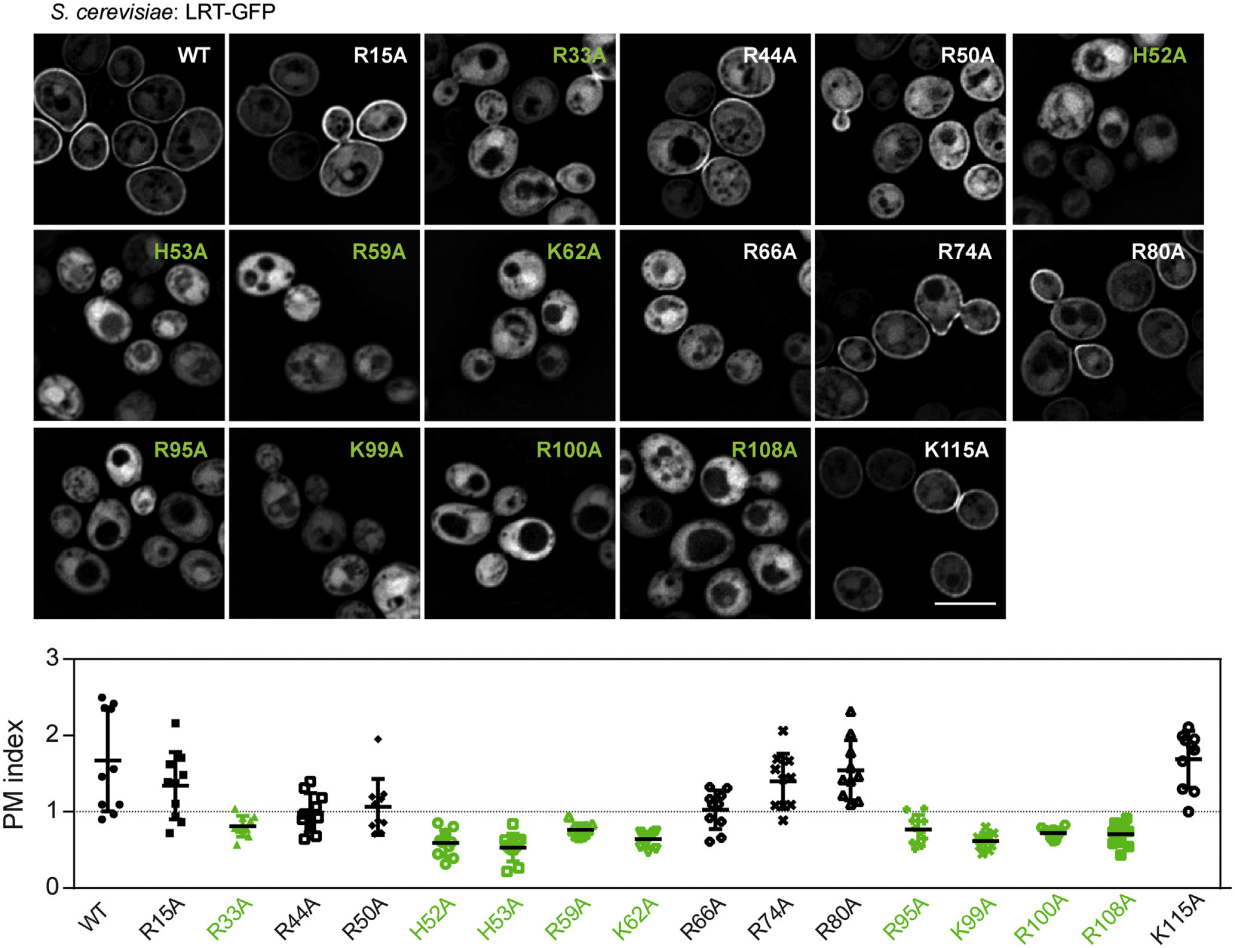
**Positively charged residues of the loop L1, helix B, and helix D are critical for the plasma membrane association of the LRT motif.***S. cerevisiae* BY4741 cells carrying plasmids encoding the indicated LRT–GFP protein variants were induced for 20 h for protein expression and examined by live-cell imaging. Representative images from two independent experiments with the same outcome are presented together with the plasma membrane (PM) index of the analyzed fusion proteins. Values of the PM index from ten randomly-selected cells expressing the indicated protein with mean ± SD are shown. See Experimental procedures for details and Table S1 for statistics. Scale bar, 5 μm. LRT, lipid raft targeting.

To gain more insight into residue specificity, we further performed glutamic acid and/or alanine mutagenesis screens of arginine, lysine, and histidine residues within the LRT motif. As determined by PM indexes of the respective variants and shown in Figure 5 and Fig. S5 and Table S1, substitutions of positively charged amino acid residues within LRT had a rather dramatic impact on PM association of the GFP-tagged LRT protein in *S. cerevisiae*. Data from both mutagenesis screens revealed that individual residues within loop L1 (H52, H53), helix B (R59, K62), and helix D (R95, K99, and R100) play a critical role in membrane interaction of LRT and/or stability of the overall structure. Besides, residue R33 within helix A and R108 within helix E were also important.

### Membrane targeting and phospholipid binding by LRT motif is dispensable for cytotoxic activity of BteA

To get a better insight into the targeting and cytotoxicity mechanisms of BteA, we next introduced the charge-reversal substitution of L1 (R50E + H52E + H53E) into the full-length protein and tested the abilities of the resulting BteA-L1 mutant to bind membrane phospholipids, localize to the PM, and induce cellular cytotoxicity.

**Figure 6:**
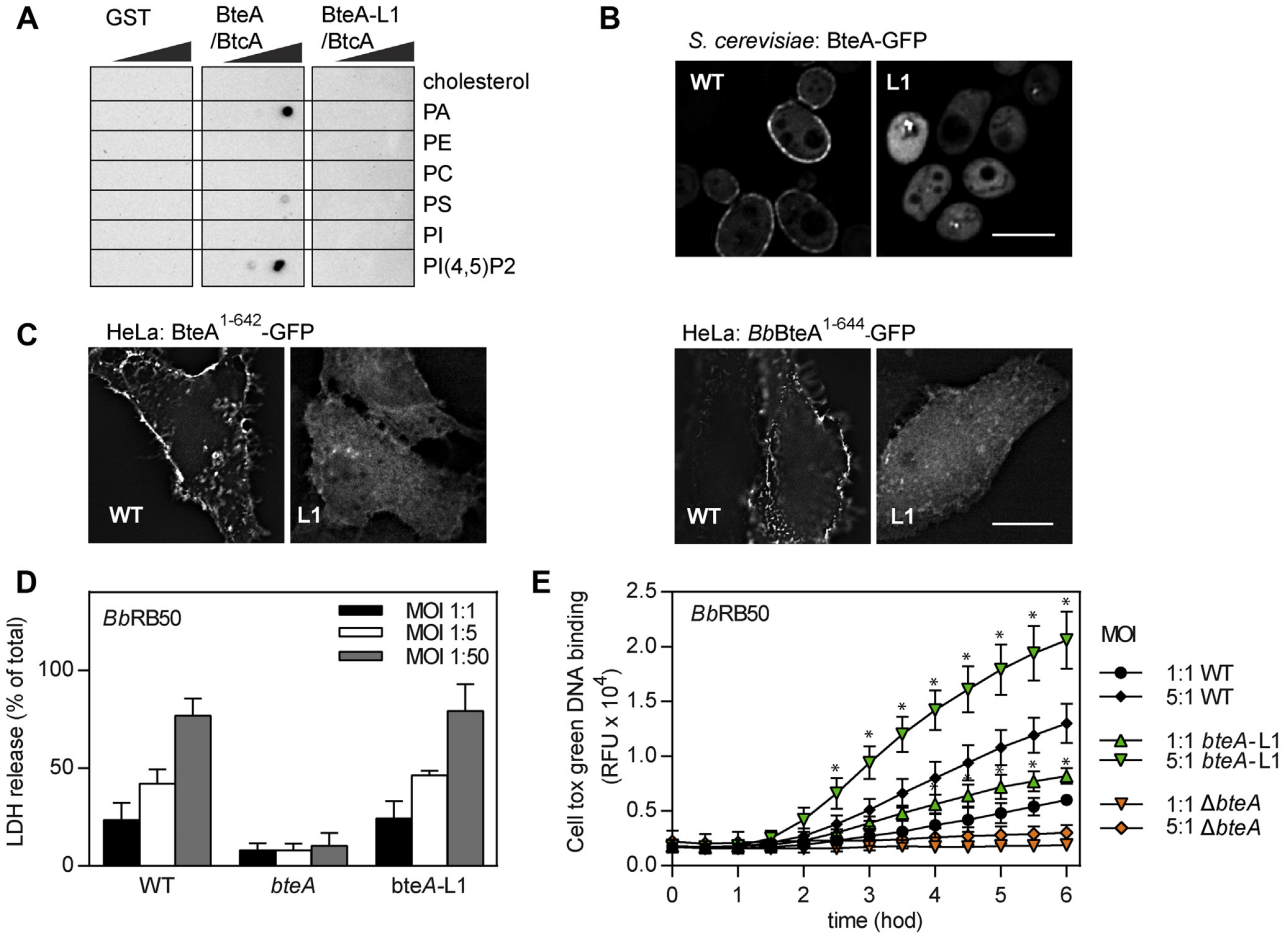
**Plasma membrane targeting of BteA effector does not contribute to its cytotoxicity.***A*, protein-lipid overlay assay. Recombinant GST-tagged full-length *B. pertussis* BteA (BteA/BtcA) and its mutated variant with charge reversal substitutions within the loop L1 (BteA-L1/BtcA; R50E + H52E + H53E) were incubated with home-made lipid arrays at 5 μg/ml. The BteA binding was detected using an anti-GST antibody, followed by chemiluminescence detection. Recombinant GST was used as a control. See the legend of Figure 1*B* for the description of spotted lipids. Results are representative of two independent experiments. *B* and *C*, localization of the GFP-tagged BteA protein variants. *B*, *S. cerevisiae* BY4741 cells harboring plasmids encoding GFP-tagged full-length BteA of *B. pertussis* and its variant BteA-GFP-L1 (L1; R50E + H52E + H53E) were cultivated for 20 h in the medium supplemented with galactose to induce protein expression. Scale bar, 5 μm. *C*, HeLa cells were transiently transfected with plasmids expressing GFP-tagged variants of *B. pertussis* BteA (BteA-1-642-GFP, BteA-1-642-L1-GFP) or *B. bronchiseptica* BteA (*Bb*BteA-1-644-GFP, *Bb*BteA-1-644-L1-GFP). After 18 h, the HeLa cells were fixed and examined by fluorescence microscopy. Scale bar, 20 μm. Representative images from two independent experiments with the same outcome are presented. *D* and *E*, HeLa cells were infected with WT, Δ*bteA*, and *bteA*-L1 mutant derivatives of *B. bronchiseptica* RB50 at the indicated multiplicity of infection (MOI). Cytotoxicity was measured as lactate dehydrogenase (LDH) release 3 h postinfection (*D*) and as real-time kinetics of membrane permeabilization determined by fluorescent DNA binding dye CellTox Green (*E*). Values represent the means ± SD from at least two independent experiments performed in duplicate (n = 4). ∗*p* < 0.05, bteA-L1 *versus* WT-infected cells, unpaired two-tailed *t* test using the corresponding infection time and MOI. GST, glutathione-S-transferase; PA, phosphatidic acid; PC, phosphatidylcholine; PE, phosphatidylethanolamine; PI, phosphatidylinositol; PS, phosphatidylserine.

The purified mutant BteA–L1 protein in complex with BtcA (BteA-L1/BtcA) was unable to bind any of the negatively charged lipids in contrast to the WT BteA/BtcA complex, binding PA, PS, and PIP2, as determined by protein–lipid overlay assay and shown in Figure 6*A*. Furthermore, the ectopically expressed mutant GFP-tagged BteA (BteA-L1) of *B. pertussis* was distributed throughout the cytoplasm of *S. cerevisiae*, whereas the WT protein localized to the cell PM (Fig. 6*B*). We were unable to visualize the full-length BteA protein of *B. bronchiseptica* in *S. cerevisiae* or BteA proteins of *B. pertussis* and *B. bronchiseptica* in transiently transfected HeLa cells because of the very high toxicity of these proteins (data not shown), as previously reported (18, 19). However, both GFP-tagged mutants of *B. pertussis* and *B. bronchiseptica* BteA without the last 14 residues (BteA^1–642^-L1 and *Bb*BteA^1–644^-L1, respectively) localized to cell cytosol in transiently transfected HeLa cells (Fig. 6*C*). In contrast, the WT variants of these truncated proteins targeted cell PM, as also shown in Figure 6*C*. Taken together, our data showed that charge-reversal substitution within loop L1 of LRT disrupted the lipid binding and membrane localization also for the full-length BteA protein.

To determine the role of PM targeting in BteA-induced cytotoxicity, the charge-reversal substitution within loop L1 (R50E + H52E + H53E, L1) was next introduced into the *bteA* open reading frame on the chromosome of *B. bronchiseptica* RB50, and the cytotoxic capacity of the mutant bacteria was tested. As shown in Figure 6*D*, the cytotoxic potency of the *bteA*-L1 mutant bacteria measured as release of the intracellular enzyme lactate dehydrogenase (LDH) into cell culture media was comparable to that of the WT strain. After 3 h of infection, both strains induced the lysis of 40% and 80% of HeLa cells at multiplicity of infection (MOI) 5:1 and 50:1, respectively. In contrast, no cell lysis was provoked by infection with Δ*bteA* strain, harboring an in-frame deletion of the BteA effector. To assess HeLa cell cytotoxicity during *B. bronchiseptica* infection in a more sensitive way, we also monitored the real-time kinetics of membrane permeabilization using a fluorescent DNA binding dye CellTox Green. Intriguingly, infection of HeLa cells by the *bteA*-L1 mutant bacteria at MOI 1:1 and 5:1 appeared to disrupt membrane integrity substantially more than the WT bacteria at the same MOI (Fig. 6*E*). Again, no membrane permeabilization was provoked by Δ*bteA* mutant strain, showing that all observed cytotoxicity of the RB50 toward HeLa cells was because of the action of the BteA effector. Taken together, these data revealed that the LRT domain does not promote BteA cytotoxicity by targeting BteA to PM of the host cells.

## Discussion

In an attempt to decipher the mechanism by which the LRT domain of *Bordetella* BteA effector targets the PM, we report here that recombinant LRT protein binds negatively-charged membrane phospholipids with a preference for PA and PIP2. Besides, we show that the domain ability to bind phospholipids and interact with membranes *in vitro* is necessary for its PM targeting in yeast and HeLa cells. Moreover, the depletion of PS and PIP2 in yeast cells mislocalizes the GFP-tagged LRT domain and full-length BteA from the PM. These data show that the association of BteA effector with the PM is guided by phospholipids PS and PIP2 *in vivo*. We also demonstrate that LRT-mediated lipid binding and PM targeting are dispensable for the cytotoxic activity of BteA effector.

Using a protein–lipid overlay assay, we showed that recombinant LRT protein encoding the MLD of BteA binds to the negatively charged phospholipids. The domain binds preferentially to PIP2 (a.k.a PI(4,5)P2) and PI(3,4)P2 as compared with PI(3,5)P2 regioisomer or various other phosphatidylinositol phosphates PI(3)P, P(4)P, P(5)P (*cf.* Fig. S1*A*). These data go well with a recent biophysical study, which detected LRT binding to PIP2-containing lipoprotein nanodiscs by shifts of NMR cross-peaks and performed NMR titration experiments using soluble phosphatidylinositol analogs (21). The lipid-binding site of the LRT domain may thus favor the presence of two phosphate groups on the inositol ring next to each other. LRT protein, however, also binds other negatively charged lipids, such as PS or PA. The LRT affinity for PIP2-containing liposomes, exhibiting Kd of ∼450 nM, is about 2.7 higher than for those enriched in PS (Kd of ∼1.2 μM) but comparable to PA-containing liposomes, which were characterized by Kd of ∼490 nM. It is worth mentioning that the liposomes that we used in our SPR experiments roughly reflected the differences between PIP2, PS, and PA abundances in cells and comprised 5% PIP2, 20% PS, or 5% PA (mol ratio) in a mixture with PC. The LRT affinities for negatively charged phospholipids are comparable to other lipid-binding domains. For example, the eukaryotic PH domain of PLCδ binds to PIP2 with Kd of ∼2 μM (25), whereas binding of full-length *Pseudomonas aeruginosa* ExoU effector to PIP2-enriched liposomes is characterized by Kd of ∼110 nM (26). Unfortunately, we could not estimate binding affinities of full-length recombinant BteA to PIP2-, PA- and PS-enriched liposomes as soluble BteA did not purify without its chaperone BtcA. This cognate chaperone binds to the LRT domain and escorts the effector for the type III secretion in *Bordetella* (27).

**Figure 7:**
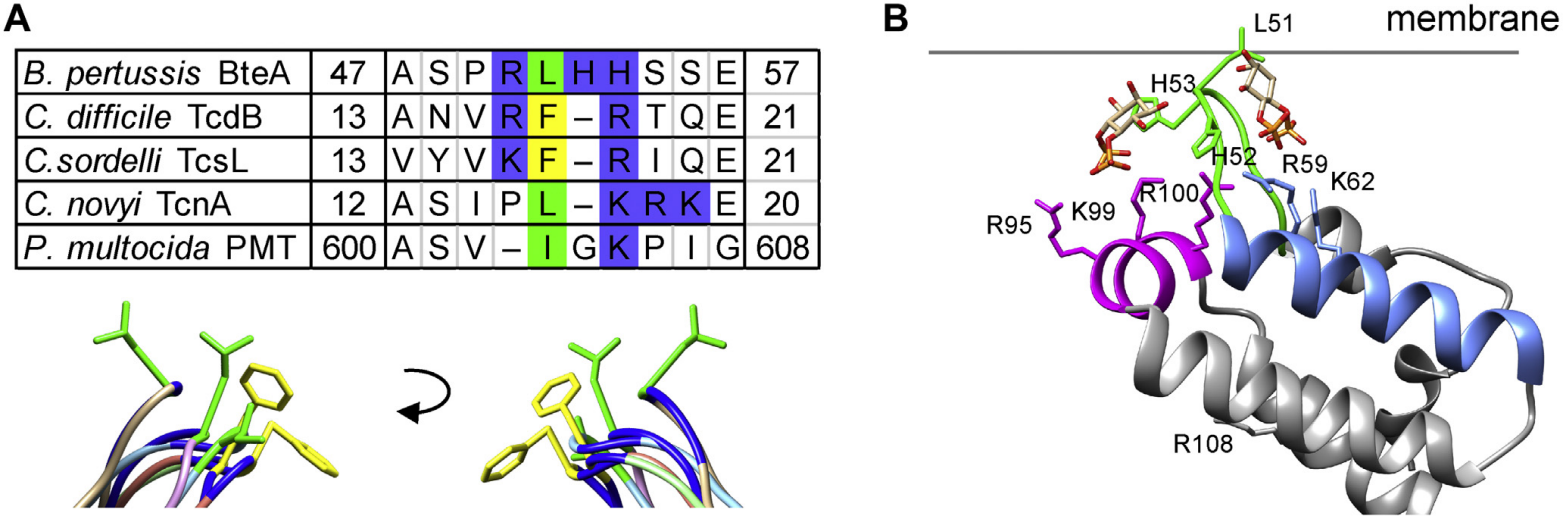
**Comparison of LRT with related PDB structures and its membrane association model.***A*, amino acid and structure alignments of the L1 region. Amino acids I/L are highlighted in *green*, F are highlighted in *yellow*, H/K/R are highlighted in *blue*. The structural alignment of the LRT domain of *B. pertussis* BteA (*brown*, PDB code: 6RGN) with 4HBM domains of *C. difficile* TcdB (*orange*, PDB code: 5UQM), *C. sordelii* TcsL (*light green*, PDB code: 2VKD), *C. novyi* TcnA (*pink*, PDB code: 2VK9), and *P. multocida* PMT (*light blue*, PDB code: 2EBF) was performed using Chimera 1.14rc. *B*, model of membrane association of the LRT domain. Critical amino acid residues identified by domain mutagenesis are depicted as *sticks* within the *ribbon* representation of the LRT structure and were visualized by Chimera 1.14rc. The headgroup of PIP2 (inositol 4,5-bisphosphate) was docked by AutoDock Vina. The binding energy was almost equal for both ligand binding sites, exhibiting the binding affinity value of −3.7 kcal/mol. LRT, lipid raft targeting.

The surface of the LRT domain structure displays a positively charged interface, which is formed by loop L1, helix B, and helix D (*cf.* Fig. 4*B*). A similar positively charged interface formed by L1 and L3 at the domain tip is present within the 4HBM domain family identified in diverse bacterial toxins (7). The 4HBM domains associate with membranes through positively charged residues of this interface in cooperation with surface-exposed hydrophobic residue at the top of loop L1 (7, 8). Importantly, surface analysis of the LRT structure pointed out an exposed hydrophobic L51 residue within loop L1 that aligns with hydrophobic residue at position 16 or 17 of 4HBM, as shown in Figure 7*A*. The L51 residue is surrounded by several basic residues, including H52 + H53, which align with conserved K/R residue at position 18 of 4HBM (Fig. 7*A*). Moreover, the substitution of the L51 residue with hydrophilic asparagine (L51N) resulted in reduced membrane binding and PM association, whereas substitution with more hydrophobic phenylalanine (L51F) increased membrane binding and PM targeting of the mutant domain variant (*cf.* Fig. 3). Besides, extensive mutagenesis of LRT residues revealed that substitutions of basic residues at the domain tip or in its proximity disrupted PM targeting of the mutant LRT variants in *S. cerevisiae*. In contrast, substitutions distant from the domain tip showed no or very little alteration (*cf.* Fig. 5 and Fig. S5 and Table S1). Our experimental data and model obtained by molecular docking of PIP2 headgroups to LRT structure (Fig. 7*B*), hence, suggest that membrane targeting by LRT is reminiscent of that of 4HBM. The L51 residue of the L1 region may directly penetrate the apolar membrane milieu, whereas the positively charged residues at the domain tip and helix B would provide a combination of electrostatic attraction to the negatively charged membrane surface and a specific headgroup recognition of the membrane phospholipids (Fig. 7*B*). Besides, helix D of LRT (Fig. 7*B*), which is analogous to L3 of 4HBM, may provide structure-stabilizing interactions. Indeed, the critical residue R100 within helix D aligns with conserved R71 residue of 4HBM and is positioned toward the structure interior. We, therefore, propose that the LRT domain of *Bordetella* BteA constitutes an additional member of the 4HBM family of bacterial proteins that targets host PM by a basic-hydrophobic motif (7, 8). The proposed LRT interaction model characterized by the membrane-penetrating leucine residue within loop L1 differs from the “side-on” interaction model suggested within the recent biophysical study (21). However, similarly to this study, we identified the critical role of residues R59 and K62 within helix B for membrane interaction of LRT (Fig. 7*B*). Further work will be needed to address the membrane targeting of homologous LRT-like domains. Importantly, we were able to locate a homologous hydrophobic leucine or isoleucine residue within the L1 loops of these domains.

The intriguing question is the differential specificity of the LRT domain of BteA as compared with the 4HBM family and the reasons for its previously reported capacity to target lipid rafts in mammalian cells (19). *In vivo*, both PIP2 and PS phospholipids were critical for PM targeting of GFP-tagged LRT domain and BteA effector in yeast cells, the model that can survive intracellular expression of the full-length BteA protein of *B. pertussis* (18). Indeed, PIP2, which constitutes only about 0.05% of the eukaryotic cell phospholipids, is highly enriched in the cytosolic leaflet of the PM (∼1% of PM phospholipids), where it controls many cell processes, including vesicle trafficking, ion channel modulation, and actin-cytoskeleton dynamics (28, 29, 30). Furthermore, depending on the cell type and experimental conditions, enrichment of PIP2 in microdomains of various sizes and/or detergent-insoluble domains has been reported (31, 32, 33, 34). PS, on the other hand, is the main negatively charged phospholipid present in the PM of eukaryotic cells, comprising ∼34% of PM phospholipids in *S. cerevisiae* and ∼8% of those in mammalian cells (35). Interestingly, PIP2 and PS were reported to have polarized localization in yeast cells, accumulating in bud necks and the bud cortex (36). It is, therefore, worth mentioning that we have noticed polarized localization of GFP-tagged LRT domain and full-length BteA during live-cell imaging of yeast cells, as shown in Fig. S6. The LRT domain exhibited continuous PM distribution and seemed to accumulate at the incipient bud site, small bud, and the mother-bud neck in the latter stages of the cell cycle. The distribution of full-length BteA did not oscillate so highly during the cell cycle and was more patch-like, suggesting additional interactions of BteA at the cell periphery compared with the LRT domain (Fig. S6). Furthermore, in contrast to LRT, BteA displayed difficulties to localize to PM in the *mss4*^*ts*^ mutant even at permissive conditions (Fig. 2*C*). In these conditions, *mss4*^*ts*^ cells have 2-fold lower levels of PIP2 as compared with the WT cells (37) and exhibit disturbance of their secretory pathways and/or actin cytoskeleton (38). This might have affected the delivery and localization of the BteA effector and/or its putative interactor. Nevertheless, despite other putative interactions of BteA, its PM targeting was entirely dependent on the LRT domain and abolished upon charge-reversal substitution within loop L1 of LRT (*cf.* Fig. 6). It remains to be established whether the LRT domain has one or more additional interacting partners other than PIP2 and PS that would further shape its specificity for lipid rafts in mammalian cells.

The LRT binding to PA might also contribute to targeting of BteA effector *in vivo.* PA is a low-abundance lipid that constitutes ∼4% of PM phospholipids in *S. cerevisiae* and ∼1% of those in mammalian cells (39, 40). It is a key intermediate in lipid metabolism and is also essential for numerous signaling pathways and membrane-sculpting processes (41). Interestingly, the LRT affinity for PA-containing liposomes (PA:PC, 5:95 molar ratio) is comparable to those enriched with PIP2 at the same molar ratio. However, determining the exact contribution of PA to PM targeting of LRT and/or full-length BteA by using live-cell imaging in yeast cells proved to be difficult (Fig. 2*B*). We have not observed any change in localization of either commonly used PA sensor GFP-Spo20^51–91^ (24) or LRT and/or full-length BteA in the *dgk1*Δ mutant of *S. cerevisiae*, which harbors a deletion of diacylglycerol kinase responsible for PA synthesis from diacylglycerol (23). Thus, no conclusions could be drawn from the performed experiments.

The mechanism of cytotoxic activity of BteA inside host cells remains unknown. Previous experiments have shown that BteA construct lacking the LRT domain displayed cell cytotoxicity indistinguishable from the full-length BteA following transient transfection (19). However, the need for LRT-dependent localization in these experiments might have been masked by the expression of BteA at high levels. By the introduction of charge-reversal substitution within loop L1 of LRT into the *bteA* open reading frame on the chromosome of *B. bronchiseptica* RB50, we found here that LRT-mediated targeting to PM does not facilitate the cytotoxic activity of BteA during bacterial infection (*cf.* Fig. 6, *D* and *E*). Moreover, at low MOI, bacteria encoding mutant BteA-L1 caused significantly more membrane permeabilization than the WT bacteria (*cf.* Fig. 6*E*). These data contrast with the requirement of membrane targeting in cytotoxicity of 4HBM-comprising bacterial toxins that have a membrane-localized target. For example, the impaired membrane localization of the *C. sordellii* TcsL mutants F17N and R18A (L1 mutations) correlated with a decrease in TscL cytotoxicity (10). Our data, hence, suggest that BteA induces cytotoxicity by a cellular pathway also initiated from the host cytoplasm. It remains to be established what is the purpose of PM localization of BteA and what kind of targets of BteA are present there. BteA localized to the PM might locally modulate host cell responses to *Bordetella* attachment (19). Indeed, *Bordetella* species colonize cilia of the polarized respiratory epithelia of diverse mammals, and BteA action within these cells might promote *Bordetella* adherence and/or subvert host cell defense by modulation of the cytoskeleton, cilia beating, and/or vesicle trafficking.

## Experimental procedures

### Bacterial and yeast strains, cell lines, media, and plasmids

All bacterial and yeast strains used in this study are listed in Tables S2 and S3, respectively, whereas the plasmids are listed in Table S4. The *E. coli* strain XL1-Blue was used for plasmid construction, *E. coli* strain Rosetta 2 was employed for recombinant protein production, and *E. coli* strain SM10 λpir was used for plasmid transfer into *Bordetella* by bacterial conjugation. *E. coli* strains were grown on Luria-Bertani agar medium or in Luria-Bertani broth, and when appropriate, media were supplemented with 100 μg/ml ampicillin and/or 30 μg/ml kanamycin. *B. bronchiseptica* strains were grown on Bordet-Gengou agar medium (Difco) supplemented with 1% glycerol and 15% defibrinated sheep blood (LabMediaServis) at 37 °C and 5% CO_2_ or in modified Stainer-Scholte medium supplemented with 5 g/l of Casamino Acids at 37 °C. Mutant *B. bronchiseptica* strains were constructed by homologous recombination using the suicide allelic exchange vector pSS4245, as described previously (18). The modified portions of the *B. bronchiseptica* chromosome were verified by sequencing (Eurofins Genomics). *S. cerevisiae* strains were grown in standard rich medium (YPD) or synthetic defined medium supplemented with appropriate amino acids and 2% glucose (SD media, noninducing media) or 2% galactose (SC-galactose media, inducing media). The media for *S. cerevisiae* BY4742 *cho1*Δ were further supplemented with 1 mM ethanolamine (Sigma Aldrich). The *S. cerevisiae* SEY6210 strains, WT, and the *mss4*^*ts*^*-102* (*mss4*^*ts*^) mutant were generous gifts of Chris Stefan. Cell lines HeLa (ATCC CCL-2, human cervical adenocarcinoma) were cultivated in Dulbecco’s Modified Eagle Medium supplemented with 10% (vol/vol) heat-inactivated fetal bovine serum at 37 °C and 5% CO_2_. The plasmids used in the study were constructed using T4 DNA ligase or the Gibson assembly strategy. PCR amplifications were performed using Herculase II Phusion DNA polymerase (Agilent) from chromosomal DNA of *B. pertussis* B1917 or *B. bronchiseptica* RB50, and PCR mutagenesis was employed to introduce the site-directed substitutions within the MLD of BteA. All constructs were verified by DNA sequencing (Eurofins Genomics). The plasmid pRS426GFP-2xPH(PLCδ) encoding the PIP2-specific probe GFP-2xPH(PLCδ) (37) was a kind gift from Chris Stefan, while the plasmids pGPD416-GFP-Lact-C2 (36) and pRS426-G20 (24) encoding the PS-specific probe GFP-Lact-C2 and the PA-specific probe GFP-Spo20^51–91^, respectively, were generously provided by Vanina Zaremberg.

### Production and purification of recombinant BteA proteins

The recombinant proteins control GST, and GST-tagged WT, and mutated variants of the full-length BteA protein (BteA, aa 1–656) of *B. pertussis* B1917 and its N-terminal MLD (LRT, aa 1–130) were produced in *E. coli* Rosetta 2 from pGEX-6P1 expression vector (GE Healthcare). To improve the solubility of the full-length protein, BteA was produced concomitantly with its chaperone BtcA expressed from the pET28b vector (Novagen) (Table S4). Exponential *E. coli* cultures grown at 30 °C were induced for protein production by the addition of IPTG to 0.1 mM at OD_600_ = 0.3 and grown for an additional 16 h at 20 °C. Bacterial cells were harvested by centrifugation, and the cell pellet was resuspended in ice-cold 50 mM Tris-HCl pH 7.4, 150 mM NaCl, and Complete Mini protease inhibitors (EDTA free, Roche). Bacterial cells were disrupted by ultrasound, and the lysate was clarified by centrifugation (30 min, 20,000*g*). The recombinant proteins were purified from the supernatant fraction using columns prepacked with Glutathione-Sepharose 4B (Amersham). The resin with bound proteins was washed with 50 mM Tris-HCl pH 7.4, 150 mM NaCl, and proteins were eluted with 10 mM reduced glutathione in 50 mM Tris-HCl pH 7.4, 150 mM NaCl. Protein preparations were dialyzed overnight into 50 mM Tris-HCl pH 7.4 and 150 mM NaCl. The integrity and purity of recombinant proteins were verified by SDS-PAGE electrophoresis followed by Coomassie blue staining, and protein concentration was determined by the Bradford protein assay.

### Protein–lipid overlay assay

Home-made lipid arrays and commercial lipid strips (cat.no. P-6002, membrane lipid strip, and cat.no. P-6001, PIP strip, both Echelon Biosciences) were used to test lipid binding of recombinant LRT and BteA proteins. To prepare home-made lipid arrays, solutions of 5 μM, 50 μM, and 500 μM of cholesterol (cat.no.C8667, Sigma Aldrich), PA (16:0–18:1; cat.no.840857P, Avanti Polar Lipids), phosphatidylethanolamine (16:0–18:1; cat.no.01991, Sigma Aldrich), PC (16:0–18:1; cat.no.42773, Sigma Aldrich), PS (16:0–18:1; cat.no.840034P, Avanti Polar Lipids), phosphatidylinositol (PI 16:0–18:1; cat.no.850142P, Avanti Polar Lipids), or phosphatidylinositol 4,5-bisphosphate (18:1–18:1; cat.no.850155P, Avanti Polar Lipids) were prepared in chloroform or in a 20:9:1 mixture of chloroform/methanol/H_2_O in the case of PIP2. Using a Hamilton syringe, 2 μl of each concentration was then spotted on the nitrocellulose membrane (BioTrace NT), yielding 10, 100, or 1000 pmol of cholesterol or lipid per spot. To analyze recombinant protein binding, the home-made arrays or commercial lipid strips (100 pmol of lipid per spot) were blocked for 1 h with PBS/0.1% (v/v) Tween-20 (PBST) containing 3% (w/v) BSA (PBST–BSA) at RT before 1 h incubation with control GST (5 μg/ml) or recombinant GST–BteA protein derivatives (5 μg/ml) in PBST–BSA. Membranes were then washed with PBST (3x 5 min), probed for 1 h with rabbit anti-GST antibody (1:2000; clone 91G1, Cell Signaling Technology) in PBST–BSA and washed again with PBST (3x 5 min). The membranes were further incubated with horseradish peroxidase–conjugated anti-rabbit IgG secondary antibody (1:3000; GE Healthcare) in PBST–BSA for 1 h. The chemiluminescence signal was detected using a Pierce ECL chemiluminescence substrate (Thermo Fisher Scientific) and an Image Quant LAS 4000 station (GE Healthcare).

### Liposome preparation

Large unilamellar liposomes/vesicles were prepared by the thin-film hydration method as previously described by (42). In brief, PC (16:0–18:1; cat.no. 42773, Sigma Aldrich) or a mixture of PS (16:0–18:1; cat.no. 840034P, Avanti Polar Lipids) with PC at a molar ratio of 20:80 (PS:PC), PA (16:0–18:1; cat.no.840857P, Avanti Polar Lipids) with PC at a molar ratio of 5:95 (PA:PC), and phosphatidylinositol 4,5-bisphosphate (18:1–18:1; cat.no. 850155P, Polar Lipids) with PC at a molar ratio of 5:95 (PIP2:PC) were dissolved in chloroform and dried in 10 × 50 mm glass test tube by rotary evaporation under a nitrogen stream. The residual solvent was removed under high vacuum for 1 h. The lipid film was hydrated at lipid:DNA ratio 1200:1 with 50 mM Tris-HCl pH 7.4, 150 mM NaCl buffer supplemented with an oligonucleotide of the sequence 5′-TATTTCTGATGTCCACCCCC-3′ modified at the 3′ end with cholesterol (Generi-Biotech). The unilamellar vesicles were prepared by extrusion of the hydrated lipid mixture through a 0.1 μm Whatman Nuclepore Track-Etched polycarbonate membrane (Sigma) assembled in a LiposoFast Basic apparatus (Avestin). The liposome suspension was supplemented with 8 mM antisense oligonucleotide of the sequence 5′-TGGACATCAGAAATACCCCC-3′ modified at the 3′ end by biotin (Generi-Biotech), and the mixture was centrifuged at 40,000*g* for 30 min at 4 °C to remove free biotinylated ssDNA. The pelleted liposomes were resuspended in 50 mM Tris-HCl pH 7.4, 150 mM NaCl, and used for binding experiments.

### Surface plasmon resonance

The SPR experiments were performed on ProteOn XPR36 Protein Interaction Array System (Bio-Rad) at 25 °C as previously described by (42). In brief, lipid vesicles (100 μg/ml) were immobilized to a neutravidin-coated NLC chip (Bio-Rad) at a flow rate of 30 μl/min to a coupling level of about 1000 resonance units. The proteins, as indicated in the figure legends, were serially diluted in the SPR running buffer, containing 50 mM Tris-HCl pH 7.4, 150 mM NaCl, and 0.005% Tween-20 and injected in parallel over the lipid surface at a constant flow rate of 30 μl/min (“one-shot kinetics”). The sensograms were corrected for sensor background by interspot referencing (the sites within the 6 × 6 array that were not exposed to ligand immobilization but were exposed to analyte flow) and double referenced by subtraction of the analyte using a “blank” injection. Assuming a Langmuir-type binding between the protein (P) and protein binding sites (S) on vesicles (*i.e.*, P + S ↔ PS), near-equilibration (R_eq_) values were then plotted against protein concentration (P_0_), and the K_D_ value was determined by nonlinear least-squares analysis of the binding isotherm using the equation R_eq_ = R_max_/(1 + K_D_/P_0_). The calculated K_D_ values represent the mean ± SD from three independent experiments.

### Yeast cell handling and live-cell fluorescence microscopy

Yeast cell transformation was carried out according to the one-step protocol of Chen *et al.* (43). The yeast centromeric vector pYC2-CT (Invitrogen) was used for galactose-inducible expression of the BteA protein variants harboring the GFP tag on their C terminus (Table S4). The *S. cerevisiae* cells carrying pYC2-CT derivatives were grown in selective glucose-containing SD media overnight and diluted into SC-galactose media (inducing media). The microscopic analysis of the cells was performed 20 h after dilution at the middle-late exponential phase of the culture. For depletion of PIP2 from the PM in *mss4*^*ts*^ strain, cells were incubated at the restrictive temperature (38 °C) for 1 h. To reduce the background fluorescence during live-cell imaging, yeast cells were washed in low-fluorescence synthetic-complete medium and mounted by covering with a thin slice of 1.5% agarose prepared in low-fluorescence synthetic-complete medium on a microscope cover glass. Widefield microscopy was performed using an Olympus IX-81 inverted microscope with 100x PlanApochromat oil-immersion objective (N.A. = 1.4) and a Hamamatsu Orca-ER-1394 digital camera. GFP fluorescence was detected with the filter block U-MGFPHQ, exc. 460 to 488 nm, em. 495 to 540 nm. Z-stacks were taken with 0.28 μm z-steps, and images were collected in a 16 bit format. Deconvolution with Advanced Maximum Likelihood filters (Xcellence Imaging SW, Olympus) and processing were performed using Olympus Cell-R Xcellence and FIJI (ImageJ) (44) software packages. Cropped images were adjusted for brightness/contrast only and mounted in Adobe Illustrator (Adobe). A single focal plane of a Z-stack is presented in all figures.

### Determination of localization score

The ability of each LRT–GFP variant to associate with yeast PM was evaluated by calculating the PM index using FIJI (ImageJ) (44) as follows. The cells were selected at random in the acquired images of different LRT-GFP variants, and the “Line tool” was used to draw the line across individual cells. The line was drawn such to avoid yeast vacuole (see yellow bars in Fig. 3*C* for demonstration), and intensities of pixels (grey values) along this line were then determined by the “Profile plot tool”. The PM index for each cell was next calculated as a ratio of the highest intensity value measured at the cell periphery and cell interior. Ten independent cells (n = 10) for each protein variant were analyzed. The significance of the differences between the WT and different LRT–GFP variants was assessed by the unpaired two-tailed *t* test.

### HeLa cell fluorescence microscopy

HeLa cells at 40% confluence on coverslips were transfected with pEGFP-N2 (Clontech) constructs encoding variants of BteA–GFP fusion protein (Table S4) using Lipofectamine 2000 reagent (Invitrogen). Eighteen hours after transfection, HeLa cells were washed with PBS and fixed by 4% formaldehyde solution in PBS (20 min, RT). The coverslips were then rinsed with distilled water and mounted onto a microscope glass slide using Vectashield mounting medium (Vector Laboratories). Fluorescence microscopy was performed using an Olympus IX-81 inverted microscope with 60x UPlanSApo oil-immersion objective (N.A. =1.35). The camera and filter set were the same as for yeast cell imaging. Z-stacks were taken with 0.32 μm z-steps, and images collected in a 16 bit format were processed as during yeast cell imaging (deconvolution with Xcellence Advanced Maximum Likelihood filters and adjustment for brightness/contrast only). A single focal plane of a Z-stack is presented in all figures.

### Preparation of cell extracts and immunoblot analysis of GFP-tagged proteins

For preparation of yeast protein extracts, yeast cells with pYC2-CT vectors encoding BteA protein derivatives (Table S4) were induced for 20 h by cultivation in SC-galactose media. Equivalents of OD_600_ = 1 of yeast cell cultures were collected, and denatured protein extracts were prepared by NaOH lysis/TCA precipitation method, according to (45). For preparation of mammalian cell protein extracts, HeLa cells at 40% confluency in a 6-well plate were transiently transfected with pEGFP-N2 vectors encoding BteA protein derivatives (Table S4) using Lipofectamine 2000 reagent (Invitrogen). Eighteen hours after transfection, cells were washed with PBS, lysed with 100 μl of ice-cold lysis buffer containing 0.2% Triton X-100 and complete mini protease inhibitors (EDTA free, Roche) in PBS, and clarified by centrifugation (5 min, 10,000*g*). Extracts were mixed with SDS-PAGE sample loading buffer, heated for 5 min at 50 °C, and separated on 10% SDS-PAGE gels. After the transfer onto nitrocellulose membrane, the proteins were probed overnight with rabbit anti-GFP antibody (1:2000; clone D5.1, Cell Signaling Technology) and revealed by horseradish peroxidase–conjugated anti-rabbit IgG secondary antibody (1:3000; GE Healthcare). Blots were developed using a Pierce ECL chemiluminescence substrate (Thermo Fisher Scientific) and an Image Quant LAS 4000 station (GE Healthcare).

### Cytotoxicity assays

Cytotoxicity of *B. bronchiseptica* RB50 strain and its derivatives (Table S2) toward HeLa cells was determined as a release of the intracellular enzyme LDH into the cell culture media using CytoTox 96 assay (cat.no. G1780, Promega) or as changes in cell membrane integrity using CellTox Green Cytotoxicity Assay (cat.no. G8743, Promega). Experiments were performed according to the manufacturer's instructions. Briefly, 2 x 10^4^ HeLa cells per well were seeded into a 96-well plate in Dulbecco’s Modified Eagle Medium supplemented with 2% (vol/vol) fetal bovine serum without the phenol red indicator and allowed to attach overnight. *B. bronchiseptica* cultures were grown to mid-exponential phase, and bacteria were added at the indicated MOI. To enable efficient infection, plates were centrifugated (5 min, 400*g*). For determination of LDH release, plates were incubated for 3 h at 37 °C and 5% CO_2_, which was followed by coupled enzymatic assay of cell culture media and absorbance measurement at 495 nm using Tecan Spark microplate reader (Tecan). The % of total LDH release was calculated using the following equation: (OD_495_ sample − OD_495_ media)/(OD_495_ total lysis − OD_495_ media) ∗ 100. For measurement of cell membrane integrity, fluorescent DNA binding dye CellTox Green was added to HeLa cells in 96-well black clear bottom plate concomitantly with *B. bronchiseptica*. The plate was then placed inside the chamber with 37 °C and 5% CO_2_ of Tecan Spark microplate reader (Tecan), and fluorescent measurement at 490ex/525em was performed in 30 min intervals for 6 h.

### Molecular docking

Molecular docking was carried out using AutoDock Vina (46). The structure of BteA–LRT (aa 29–121, PDB code: 6RGN) was processed by removing all sulfate ions and adding all hydrogens using AutoDockTools. The protein molecule was considered as a rigid molecule, whereas the ligand (inositol 4,5-bisphosphate) was treated as being flexible. The binding site region was defined as a region, which encompasses the entire surface of the protein. The binding energy was almost equal for both ligand binding sites, with the binding affinity value of −3.7 kcal/mol. The results of the docking were visualized using Chimera 1.14rc (47) and are shown in Figure 7*B*.

## Data availability

All data are contained within this article and in the supporting information.

## Supporting information

This article contains supporting information (48, 49, 50, 51, 52, 53, 54, 55, 56, 57).

## Conflict of interest

The authors declare that they have no conflicts of interest with the contents of this article.
