# Supplementary material for: Lipid binding by the N-terminal motif mediates plasma membrane localization of Bordetella effector protein BteA
Source: J Biol Chem. 2021 Mar 28;296:100607. doi: 10.1016/j.jbc.2021.100607 (PMC8100071; doi:10.1016/j.jbc.2021.100607)
Supplement: Supplemental Figures S1–S6 and Tables S1–S4 [file mmc1.pdf]

Supporting information for

Lipid binding by the N-terminal motif mediates plasma membrane localization of *Bordetella* effector protein BteA

Ivana Malcova[1‡], Ladislav Bumba[1‡], Filip Uljanic[1], Darya Kuzmenko[1], Jana Nedomova[1] and Jana Kamanova[1*]

[1]Institute of Microbiology of the Czech Academy of Sciences, Prague, Czech Republic

Running title: Interaction of LRT domain of BteA with plasma membrane

‡ These authors contributed equally to this work.
* For correspondence: Jana Kamanova, kamanova@biomed.cas.cz

This supporting information includes:

Supplementary Figure S1. Phospholipid binding by the N-terminal motif of *Bordetella* effector BteA.

Supplementary Figure S2. Phospholipids PS and PIP2 guide plasma membrane association of *Bordetella* BteA effector and its LRT motif in cells.

Supplementary Figure S3. Leu51 residue is involved in hydrophobic interactions of the LRT motif with a phospholipid membrane.

Supplementary Figure S4. Charge-reversal substitutions within the LRT segment do not affect GFP-fusion protein stability in yeast and HeLa cells.

Supplementary Figure S5. Positively charged residues of the loop L1, helix B, and helix D are critical for the plasma membrane association of the LRT motif.

Supplementary Figure S6. *Bordetella* BteA effector and its LRT domain exhibit a preferential polarized localization in yeast cells.

Supplementary Table S1. Alanine and glutamic acid mutagenesis of positively charged amino acid residues in the LRT motif of BteA.

Supplementary Table S2. List of bacterial strains used in this study.

Supplementary Table S3. List of yeast strains and mammalian cell lines used in this study.

Supplementary Table S4. List of plasmids used in this study.

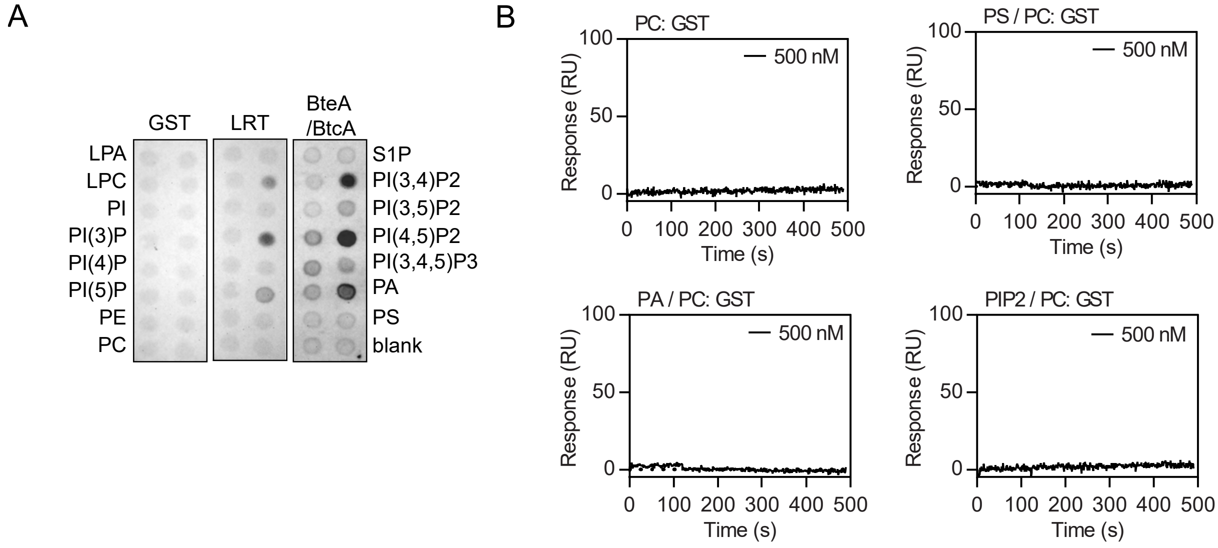

Figure S1. Phospholipid binding by the N-terminal motif of *Bordetella* effector BteA.
(A) Protein-lipid overlay assay. The recombinant GST-tagged N-terminal LRT domain (LRT) and full-length BteA (BteA/BtcA) protein of *B. pertussis* were incubated at 5 µg/ml with commercial lipid strips. The binding was detected using an anti-GST antibody followed by chemiluminescence detection. Recombinant GST was used as a control. Lysophosphatidic acid (LPA); lysophophocholine (LPC); phosphatidylinositol (PI); phosphatidylinositol phosphates (PIP, PIP2, PIP3); phosphatidylethanolamine (PE); phosphatidylcholine (PC); sphingosine-1-phosphate (S1P); phosphatidic acid (PA), and phosphatidylserine (PS).
B) SPR kinetic binding analyses of the interaction between GST and lipid vesicles. Serially diluted GST protein (at 500, 250, 125, 62.5, and 31.25 nM concentrations) was injected in parallel over the neutravidin sensor chip coated with the immobilized liposomes (100 nm in diameter) containing PC, PS/PC (20:80), PA/PC (5:95), or PIP2/PC (5:95), and left to associate (120 s) and dissociate (380 s) at constant flow rate of 30 µl/min. For clarity, only the binding curve for the highest concentration of GST (500 nM) is shown. The sensograms show the representative binding curves from three independent “one-shot kinetic” experiments.

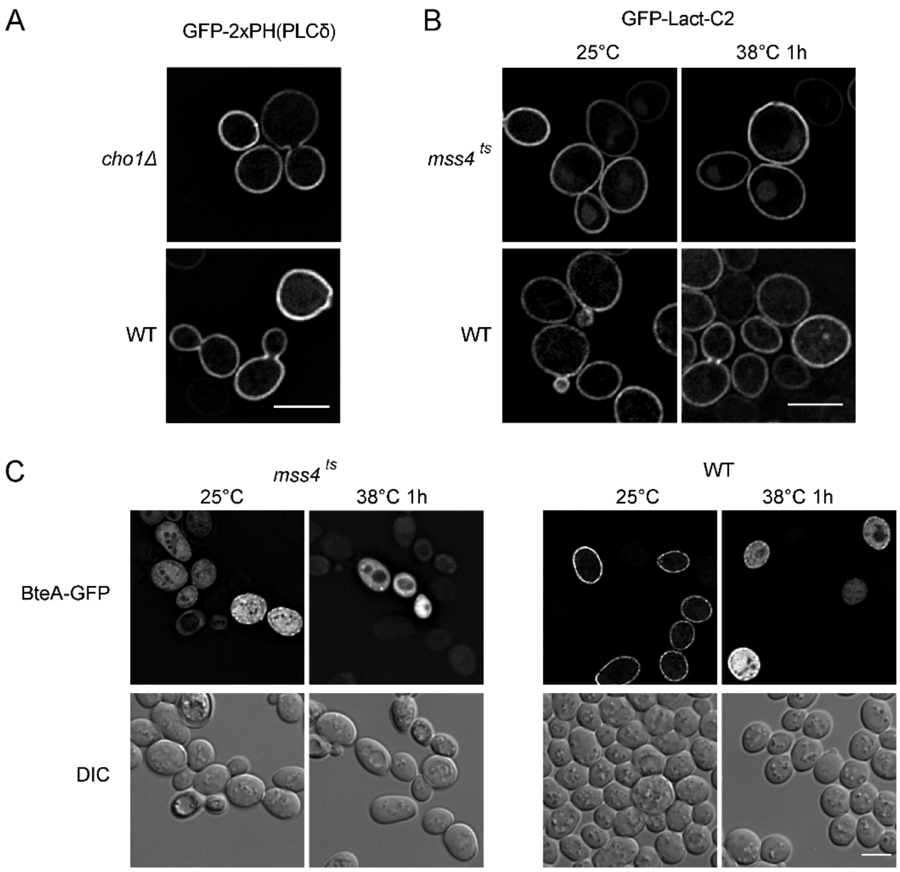

Figure S2. Phospholipids PS and PIP2 guide plasma membrane association of *Bordetella* BteA effector and its LRT motif in cells.

(A-B) Phospholipid levels in the yeast cells were monitored by localization of GFP-tagged lipid-specific probes. **(A)** PIP2-specific probe 2xPH(PLCδ) was used as a control of the specificity of decreased PS levels in the *cho1*Δ derivative of the *S. cerevisiae* BY4742, whereas **(B)** PS-specific probe GFP-Lact-C2 monitored the specificity of decreased PIP2 levels in the $mss4^{ts}$ of *S. cerevisiae* SEY6210 at the restrictive temperature. Representative images from two independent experiments with the same outcome are presented. Scale bar, 5 μm.

(C) GFP-tagged BteA (BteA-GFP) effector of *B. pertussis* was visualized upon galactose induction in the temperature-sensitive $mss4^{ts}$ mutant and wild type (WT) strain of *S. cerevisiae* SEY6210 after the shift from the permissive (25 °C) to restrictive temperature (38 °C). Representative images from two independent experiments with the same outcome are presented. Scale bar, 5 μm.

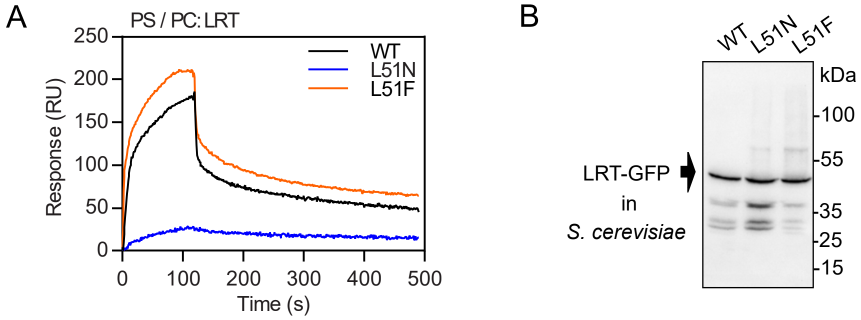

Figure S3. Leu51 residue is involved in hydrophobic interactions of the LRT motif with a phospholipid membrane.

(A) Overlay plot of SPR sensograms obtained after injection of LRT, LRT-L51N, and LRT-L51F proteins at 250 nM concentration over the neutravidin sensor chip coated with the immobilized PS/PC (20:80) lipid vesicles. The binding curves are representative of five independent "one-shot kinetic" experiments.

(B) Western blot analysis. Protein extracts were prepared from yeast cell cultures with plasmids encoding the indicated GFP-tagged LRT protein variants after 20 h induction with galactose. Equal volumes of extracts (0.4 ml of the culture equivalent; OD600 = 1) were separated on SDS-PAGE and analyzed by immunoblot using an anti-GFP antibody (1: 2,000). The arrow indicates the molecular weight of the intact LRT-GFP fusion protein.

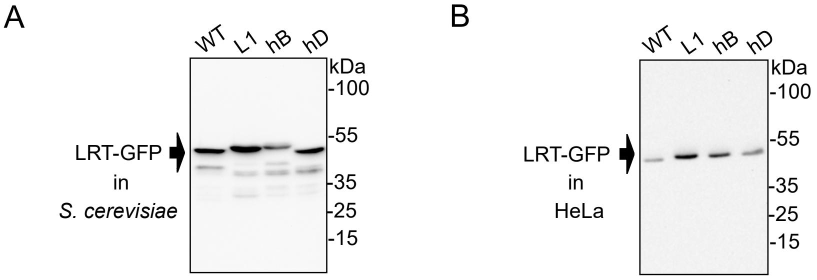

Figure S4. Charge-reversal substitutions within the LRT segment do not affect GFP-fusion protein stability in yeast and HeLa cells.
Protein extracts were prepared from **(A)** yeast cell cultures expressing the indicated GFP-tagged LRT protein variants after 20 h induction with galactose or **(B)** HeLa cells 18 h post-transfection. The equal volumes of extracts were separated on SDS-PAGE and analyzed by immunoblot using an anti-GFP antibody (1: 2,000). The arrow indicates the molecular weight of the intact LRT-GFP fusion protein.

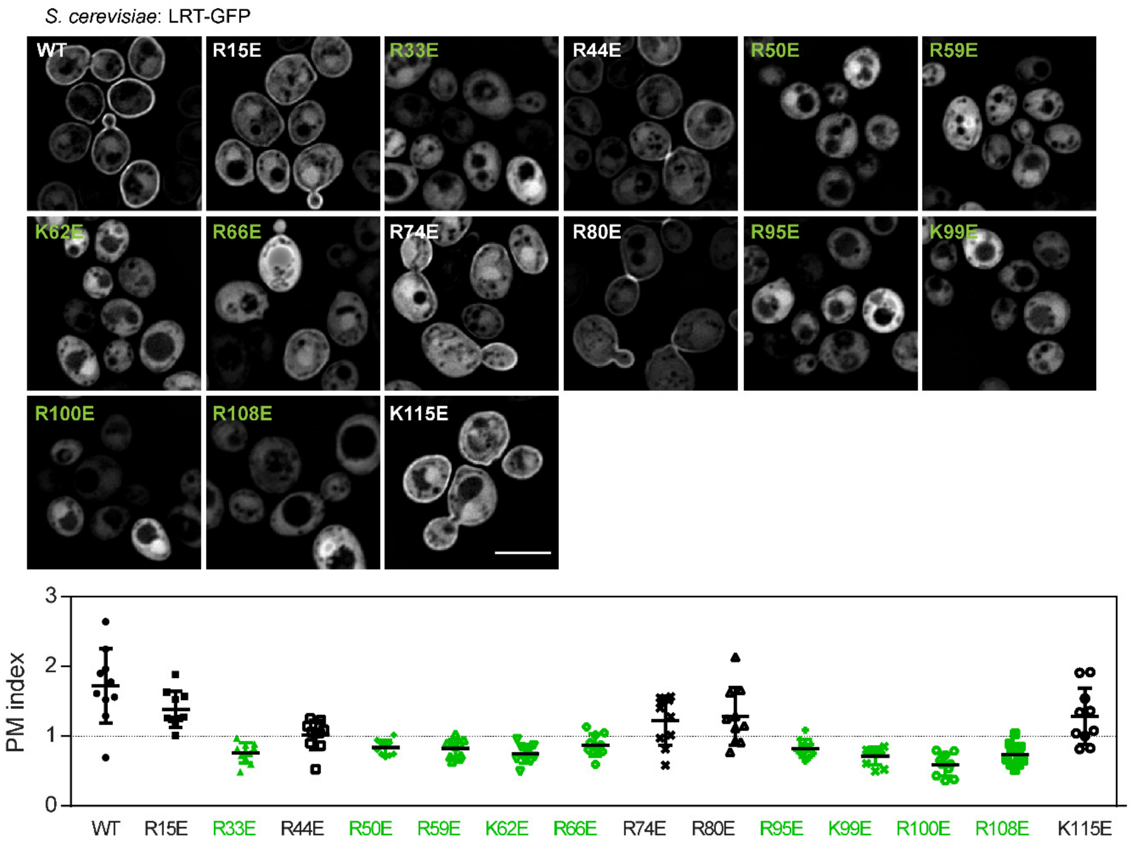

Figure S5. Positively charged residues of the loop L1, helix B, and helix D are critical for the plasma membrane association of the LRT motif.

S. cerevisiae BY4741 cells carrying plasmids encoding the indicated LRT-GFP protein variants were induced for 20 h for protein expression and examined by live-cell imaging. Representative images from two independent experiments with the same outcome are presented together with the plasma membrane (PM) index of the analyzed fusion proteins. Values of the PM index from 10 randomly-selected cells expressing the indicated protein with mean ± SD are shown. See Experimental procedures for details and Table S1 for statistics. Scale bar, 5 µm.

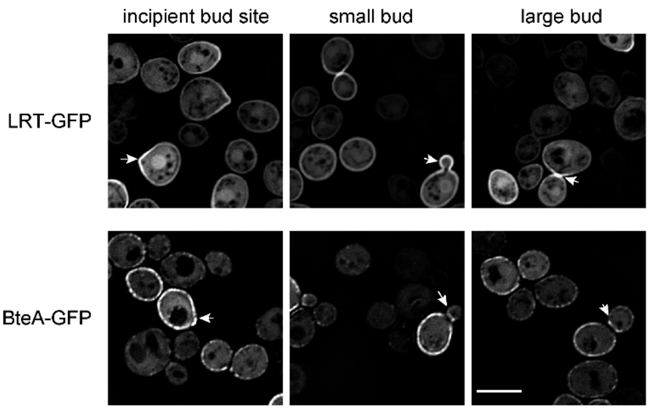

Figure S6. ***Bordetella*** **BteA effector and its LRT domain exhibit a preferential polarized localization in yeast cells.**
S. cerevisiae BY4741 harboring plasmids encoding GFP-tagged LRT domain (LRT-GFP) or full-length BteA (BteA-GFP) were cultivated for 20 h in the medium supplemented with galactose to induce protein expression followed by their live-cell imagining. Arrows in respective panels point to incipient bud sites, small buds and mother-bud necks of large buds. Scale bar, 5μm.

Table S1. Alanine and glutamic acid mutagenesis of positively charged amino acid residues in the LRT motif of BteA. The cellular distribution of the indicated variants of LRT-GFP fusion proteins in *S. cerevisiae* was evaluated using intensity profile plots. See Experimental procedures for details. The mean ± SD of plasma membrane (PM) index from 10 cells (n=10) expressing the respective LRT variant is presented. The significance of differences was tested by unpaired two-tailed t-test as compared to LRT-WT. The significance levels are indicated as follows: ns, not significant; *, p< 0.01; **, p<0.001; ***, p<0.0001; ****, p<0.00001. The protein variants with a significance level of p< 0.001 are highlighted in green. ND, not determined.

Alanine mutagenesis			Glutamic acid mutagenesis		
Substitution	PM index (mean ± SD)	Significance level	Substitution	PM index (mean±SD)	Significance level
WT	1.7 ± 0.6		WT	1.7 ± 0.5	
R15A	1.3 ± 0.4	*ns*	R15E	1.4 ± 0.2	*ns*
R33A	0.8 ± 0.1	**	R33E	0.8 ± 0.1	***
R44A	1.0 ± 0.2	*	R44E	1.0 ± 0.2	*
R50A	1.0 ± 0.3	*ns*	R50E	0.8 ± 0.1	***
H52A	0.6 ± 0.2	***	H52E	ND	ND
H53A	0.5 ± 0.2	***	H53E	ND	ND
R59A	0.8 ± 0.1	**	R59E	0.8 ± 0.1	***
K62A	0.6 ± 0.1	**	K62E	0.7 ± 0.1	***
R66A	1.0 ± 0.2	*ns*	R66E	0.9 ± 0.2	**
R74A	1.4 ± 0.3	*ns*	R74E	1.2 ± 0.3	*ns*
R80A	1.5 ± 0.4	*ns*	R80E	1.3 ± 0.4	*ns*
R95A	0.8 ± 0.2	**	R95E	0.8 ± 0.1	***
K99A	0.6 ± 0.1	**	K99E	0.7 ± 0.1	***
R100A	0.7 ± 0.1	**	R100E	0.6 ± 0.2	****
R108A	0.7 ± 0.1	**	R108E	0.7 ± 0.2	***
K115A	1.7 ± 0.4	*ns*	K115E	1.3 ± 0.4	*ns*

Table S2. List of bacterial strains used in this study. Bacterial strain name, genotype description, and reference are indicated.

Strain	Genotype and relevant description	Reference
Escherichia coli strains		
XL1-Blue	*rec*A1 *end*A1 *gyr*A96 *thi*-1 *hsd*R17 *sup*E44 *rel*A1 *lac* F′ *pro*AB l*ac*IqZΔM15 Tn10 Tet^r	Stratagene, USA
Rosetta 2	F^- *ompT hsdS*$_B$(r_B^- m_B^-) *gal dcm* (DE3) pRARE2 (Cam^R), BL21 derivatives designed to enhance the expression of proteins that contain codons rarely used in *E. coli*	Novagen, USA
SM10 λpir	*thi thr leu tonA lacY supE recA:*:RP4-2-Tc::Mu Km λ*pir*	(48,49)
Bordetella pertussis strains		
*Bp*B1917	wild type *Bordetella pertussis* B1917*; fim2-1, fim3-2, ptxP3, ptxA1, ptxB2, ptxC2, ptxD1, ptxE1, prn2*	(50,51)
Bordetella bronchiseptica strains		
WT	*Bb*RB50; wild type *Bordetella bronchiseptica* RB50 (B1976); complex I rabbit isolate; ST-12	(52,53)
Δ*bteA*	*Bb*RB50 Δ*bteA; Bb*RB50 strain derivative with *bteA* in-frame deletion of codons L2-A657	this study
bteA-L1	*BbRB50 bteA*-L1*; Bb*RB50 strain derivative encoding mutated *bteA* allele with 3 codon substitutions R50E+H52E+H53E	this study

Table S3. List of yeast strains and mammalian cell lines used in this study. Names, descriptions, and references are indicated.

	**Genotype and relevant description**	**Reference**
Saccharomyces cerevisiae strains		
BY4741	*MAT*a; *his3*Δ1; *leu2*Δ0; *ura3*Δ0; *met15*Δ0	(54), Euroscarf, Germany
BY4741 *dgk1*Δ	*MAT*a; *his3*Δ1; *leu2*Δ0; *ura3*Δ0; *met15*Δ0; *dgk1::kan*MX4	Euroscarf, Germany
BY4742	*MAT*α; *his3*Δ1; *leu2*Δ0; *ura3*Δ0; *lys2*Δ0	(54), Euroscarf, Germany
BY4742 *cho1*Δ	*MAT*α; *his3*Δ1; *leu2*Δ0; *ura3*Δ0; *lys2*Δ0; *cho1::kan*MX4	Euroscarf, Germany
SEY6210	*MAT*α; *leu2-3,112; ura3-52; his3-Δ200; trp1-Δ901; lys2-801; suc2-Δ9*	(55)
SEY6210 *mss4^{ts}*	*MAT*α; *leu2-3,112; ura3-52; his3-Δ200; trp1-Δ901; lys2-801; suc2-Δ9; mss4Δ::HIS3*MX6 + YC*plac*111*mss4^{ts}-102* [*LEU2 CEN6 mss4^{ts}-102*]	(37)
Mammalian cell lines		
HeLa	Human cervical adenocarcinoma cell line	ATCC, CCL-2™

Table S4. List of plasmids used in this study. Plasmid names, descriptions, and references are provided.

**Plasmid**	**Description**	**Reference**
pET28b	His-tagging expression vector for *E. coli*, T7 promoter, lac operator, Km^R	Novagen, USA
pET28b-BtcA	pET28b vector encoding the BtcA chaperone of *Bp*B1917 fused with 6xHis tag on its N-terminus	this study
pGEX-6P1	GST-tagging expression vector for *E. coli*, lac operator, Amp^R	GE Healthcare, USA
pGEX-6P1-LRT	pGEX-6P1 vector encoding the LRT domain (aa 1-130) of BteA effector of *Bp*B1917 fused with GST on its N-terminus	this study
pGEX-6P1-LRT-L51N	pGEX-6P1-LRT harboring the LRT-L51N substitution	this study
pGEX-6P1-LRT-L51F	pGEX-6P1-LRT harboring the LRT-L51F substitution	this study
pGEX-6P1-LRT-L1	pGEX-6P1-LRT harboring the LRT-R50E+H52E+H53E substitutions	this study
pGEX-6P1-LRT-hB	pGEX-6P1-LRT harboring the LRT-R59E+K62E+R66E substitutions	this study
pGEX-6P1-LRT-hD	pGEX-6P1-LRT harboring the LRT-K99E+R100E substitutions	this study
pGEX-6P1-BteA	pGEX-6P1 vector encoding the BteA effector of *Bp*B1917 fused with GST on its N-terminus	this study
pGEX-6P1-BteA-L1	pGEX-6P1-BteA harboring the LRT-R50E+H52E+H53E substitutions	this study
pYC2-CT	An expression vector for *S. cerevisiae*, *GAL1* promoter, *URA3* marker, Amp^R, *CEN6*/ARS4	Invitrogen
pYC2-CT-LRT-GFP	pYC2-CT vector encoding the LRT domain (aa 1-130) of BteA effector of *Bp*B1917 fused with GFP on its C-terminus, the gene is under the control of the *GAL1* promoter	this study
pYC2-CT-LRT-L51N-GFP	pYC2-CT-LRT-GFP harboring the LRT-L51N substitution	this study
pYC2-CT-LRT-L51F-GFP	pYC2-CT-LRT-GFP harboring the LRT-L51F substitution	this study
pYC2-CT-LRT-L1-GFP	pYC2-CT-LRT-GFP harboring the LRT-R50E+H52E+H53E substitutions	this study

pYC2-CT-LRT-hB-GFP	pYC2-CT-LRT-GFP harboring the LRT-R59E+K62E+R66E substitutions	this study
pYC2-CT-LRT-hD-GFP	pYC2-CT-LRT-GFP harboring the LRT-K99E+R100E substitutions	this study
Constructs of pYC2-CT-LRT-GFP used in a glutamic acid mutagenesis screen	Set of pYC2-CT-LRT-GFP harboring the following substitutions within LRT: R15E, R33E, R44E, R50E, R59E, K62E, R66E, R74E, R80E, R95E, K99E, R100E, R108E, K115E	this study
Constructs of pYC2-CT-LRT-GFP used in an alanine mutagenesis screen	Set of pYC2-CT-LRT-GFP harboring the following substitutions within LRT: R15A, R33A, R44A, R50A, H52A, H53A, R59A, K62A, R66A, R74A, R80A, R95A, K99A, R100A, R108A, K115A	this study
pYC2-CT-BteA-GFP	pYC2-CT vector encoding the BteA effector of *Bp*B1917 fused with GFP on its C-terminus, the gene is under the control of the *GAL1* promoter	(18)
pYC2-CT-BteA-L1-GFP	pYC2-CT-BteA-GFP harboring the LRT-R50E+H52E+H53E substitutions	this study
pGPD416-GFP-Lact-C2	PS-specific probe, *GPD* promoter, *URA3* marker, Amp^R, *CEN6*/ARS4, GFP-Lact-C2	(36)
pRS426GFP-2xPH(PLCδ)	PI(4,5)P2-specific probe, *CPY* promoter, *URA3* marker, Amp^R, 2μm, GFP-2xPH(PLCδ)	(37)
pRS426-G20	PA-specific probe, *TEF*2 promoter, *URA3* marker, Amp^R, 2μm, GFP-$Spo20^{51\text{-}91}$	(24)
pEGFPN2	GFP-tagging mammalian expression vector, immediate early promoter of CMV, Km^R	Clontech, USA
pEGFPN2-LRT-GFP	pEGFPN2 vector encoding the LRT domain (aa 1-130) of BteA effector of *Bp*B1917 fused with GFP on its C-terminus	this study
pEGFPN2-LRT-L1-GFP	pEGFPN2-LRT-GFP harboring the LRT-R50E+H52E+H53E substitutions	this study
pEGFPN2-LRT-hB-GFP	pEGFPN2-LRT-GFP harboring the LRT-R59E+K62E+R66E substitutions	this study
pEGFPN2-LRT-hD-GFP	pEGFPN2-LRT-GFP harboring the LRT-K99E+R100E substitutions	this study
pEGFPN2-BteA-1-642-GFP	pEGFPN2 vector encoding BteA effector of *Bp*B1917 without its last 14 aa residues (aa 1-642) fused with GFP on its C-terminus	this study
pEGFPN2-BteA-1-642-L1-GFP	pEGFPN2-BteA-1-642-GFP harboring the LRT-R50E+H52E+H53E substitutions	this study

pEGFPN2-*Bb*BteA-1-644-GFP	pEGFPN2 vector encoding BteA effector of *Bb*RB50 without its last 14 aa residues (aa 1-644) fused with GFP on its C-terminus	this study
pEGFPN2-*Bb*BteA-1-644-L1-GFP	pEGFPN2-*Bb*BteA-1-644-GFP harboring the LRT-R50E+H52E+H53E substitutions	this study
pSS4245	An allelic exchange vector for *Bordetella spp.*, contains *ptx* promoter*, I-SceI*, *oriV*, *AmpR, StrR, KmR, BleR, TetR* and an *I-SceI* cleavage site for counterselection	(56,57)
pSS4245-*Bb*RB50-*ΔbteA*	pSS4245 vector containing homology regions h1 (712 bp, 4501345-4502056) and h2 (622 bp, 4504028-4504649) flanking in-frame deletion of codons L2-A657 in the *bteA* gene of *Bb*RB50	this study
pSS4245-*Bb*RB50-*bteA*-L1	pSS4245 vector containing homology regions h1 (692 bp, 4501509-4502200) and h2 (683 bp, 4502213-4502895) flanking in-frame substitutions of codons R50E+H51E+H52E in the *bteA* gene of *Bb*RB50	this study

Supporting References:

18. Bayram, J., Malcova, I., Sinkovec, L., Holubova, J., Streparola, G., Jurnecka, D., Kucera, J., Sedlacek, R., Sebo, P., and Kamanova, J. (2020) Cytotoxicity of the effector protein BteA was attenuated in Bordetella pertussis by insertion of an alanine residue. *Plos Pathog* **16**, e1008512
24. Nakanishi, H., de los Santos, P., and Neiman, A. M. (2004) Positive and negative regulation of a SNARE protein by control of intracellular localization. *Mol Biol Cell* **15**, 1802-1815
36. Fairn, G. D., Hermansson, M., Somerharju, P., and Grinstein, S. (2011) Phosphatidylserine is polarized and required for proper Cdc42 localization and for development of cell polarity. *Nat Cell Biol* **13**, 1424-1430
37. Stefan, C. J., Audhya, A., and Emr, S. D. (2002) The yeast synaptojanin-like proteins control the cellular distribution of phosphatidylinositol (4,5)-bisphosphate. *Mol Biol Cell* **13**, 542-557
48. Simon, R., Priefer, U., and Pühler, A. (1983) A Broad Host Range Mobilization System for In Vivo Genetic Engineering: Transposon Mutagenesis in Gram Negative Bacteria. *Bio/Technology* **1**, 784
49. Skopova, K., Tomalova, B., Kanchev, I., Rossmann, P., Svedova, M., Adkins, I., Bibova, I., Tomala, J., Masin, J., Guiso, N., Osicka, R., Sedlacek, R., Kovar, M., and Sebo, P. (2017) Cyclic AMP-Elevating Capacity of Adenylate Cyclase Toxin-Hemolysin Is Sufficient for Lung Infection but Not for Full Virulence of Bordetella pertussis. *Infect Immun* **85**
50. Bart, M. J., Zeddeman, A., van der Heide, H. G., Heuvelman, K., van Gent, M., and Mooi, F. R. (2014) Complete Genome Sequences of Bordetella pertussis Isolates B1917 and B1920, Representing Two Predominant Global Lineages. *Genome Announc* **2**
51. Bart, M. J., Harris, S. R., Advani, A., Arakawa, Y., Bottero, D., Bouchez, V., Cassiday, P. K., Chiang, C. S., Dalby, T., Fry, N. K., Gaillard, M. E., van Gent, M., Guiso, N., Hallander, H. O., Harvill, E. T., He, Q., van der Heide, H. G., Heuvelman, K., Hozbor, D. F., Kamachi, K., Karataev, G. I., Lan, R., Lutynska, A., Maharjan, R. P., Mertsola, J., Miyamura, T., Octavia, S., Preston, A., Quail, M. A., Sintchenko, V., Stefanelli, P., Tondella, M. L., Tsang, R. S., Xu, Y., Yao, S. M., Zhang, S., Parkhill, J., and Mooi, F. R. (2014) Global population structure and evolution of Bordetella pertussis and their relationship with vaccination. *MBio* **5**, e01074
52. Cotter, P. A., and Miller, J. F. (1994) BvgAS-mediated signal transduction: analysis of phase-locked regulatory mutants of Bordetella bronchiseptica in a rabbit model. *Infect Immun* **62**, 3381-3390
53. Diavatopoulos, D. A., Cummings, C. A., Schouls, L. M., Brinig, M. M., Relman, D. A., and Mooi, F. R. (2005) Bordetella pertussis, the causative agent of whooping cough, evolved from a distinct, human-associated lineage of B. bronchiseptica. *Plos Pathog* **1**, e45
54. Brachmann, C. B., Davies, A., Cost, G. J., Caputo, E., Li, J., Hieter, P., and Boeke, J. D. (1998) Designer deletion strains derived from Saccharomyces cerevisiae S288C: a useful set of strains and plasmids for PCR-mediated gene disruption and other applications. *Yeast* **14**, 115-132
55. Robinson, J. S., Klionsky, D. J., Banta, L. M., and Emr, S. D. (1988) Protein sorting in Saccharomyces cerevisiae: isolation of mutants defective in the delivery and processing of multiple vacuolar hydrolases. *Mol Cell Biol* **8**, 4936-4948
56. Inatsuka, C. S., Xu, Q., Vujkovic-Cvijin, I., Wong, S., Stibitz, S., Miller, J. F., and Cotter, P. A. (2010) Pertactin is required for Bordetella species to resist neutrophil-mediated clearance. *Infect Immun* **78**, 2901-2909
57. Posfai, G., Kolisnychenko, V., Bereczki, Z., and Blattner, F. R. (1999) Markerless gene replacement in Escherichia coli stimulated by a double-strand break in the chromosome. *Nucleic Acids Res* **27**, 4409-4415
